# Exploratory analyses on the effect of time since last meal on concentrations of amino acids, lipids, one-carbon metabolites, and vitamins in the Hordaland Health Study

**DOI:** 10.1007/s00394-023-03211-y

**Published:** 2023-07-27

**Authors:** Åslaug Matre Anfinsen, Hanne Rosendahl-Riise, Ottar Nygård, Grethe Seppola Tell, Per Magne Ueland, Arve Ulvik, Adrian McCann, Jutta Dierkes, Vegard Lysne

**Affiliations:** 1 Mohn Nutrition Research Laboratory, Centre for Nutrition, Department of Clinical Science, University of Bergen, Bergen, Norway; 2 Mohn Nutrition Research Laboratory, Centre for Nutrition, Department of Clinical Medicine, University of Bergen, Bergen, Norway; 3 Department of Heart Disease, Haukeland University Hospital, Bergen, Norway; 4 Department of Global Public Health, University of Bergen, Bergen, Norway; 5 Bevital, Bergen, Norway; 6 Department of Laboratory Medicine and Pathology, Haukeland University Hospital, Bergen, Norway

**Keywords:** Biomarkers, Metabolites, Categorization, Fasting, Postprandial, Epidemiology

## Abstract

**Purpose:**

Dietary intake may have pronounced effects on circulating biomarker concentrations. Therefore, the aim was to provide a descriptive overview of serum metabolite concentrations in relation to time since last meal, focusing on amino acids, lipids, one-carbon metabolites, and biomarkers of vitamin status.

**Methods:**

We used baseline data from the observational community-based Hordaland Health Study, including 2960 participants aged 46–49 years and 2874 participants aged 70–74 years. A single blood draw was taken from each participant, and time since last meal varied. Estimated marginal geometric mean metabolite concentrations were plotted as a function of time since last meal, up to 7 h, adjusted for age, sex, and BMI.

**Results:**

We observed a common pattern for nearly all amino acids and one-carbon metabolites with highest concentrations during the first 3 h after dietary intake. Homocysteine and cysteine were lowest the 1st hour after a meal, while no patterns were observed for glutamate and glutamic acid. The concentrations of phylloquinone and triglycerides were highest 1 h after dietary intake. Thiamine and thiamine monophosphate concentrations were highest, while flavin mononucleotide concentrations were lowest within the first 2 h after a meal. No clear patterns emerged for the other fat-soluble vitamins, blood lipids, or B-vitamin biomarkers.

**Conclusion:**

Our findings suggest that distinguishing between “fasting” and “non-fasting” blood samples may be inadequate, and a more granular approach is warranted. This may have implications for how to account for dietary intake when blood sampling in both clinical and research settings.

**Supplementary Information:**

The online version contains supplementary material available at 10.1007/s00394-023-03211-y.

## Introduction

Blood metabolites are frequently used in epidemiological studies to assess the relationship with an incident disease or death. However, there are many sources of measurement error in metabolites when they are used in epidemiological studies. These measurement errors do not only encompass laboratory errors but may also arise from within-person variability. Variability in metabolite concentrations within subjects can be short term and caused by factors such as circadian rhythm and dietary intake, or long term caused by seasonal changes in diet or transient illness. As metabolite concentrations may fluctuate and change within subjects, a single measurement may inadequately capture the true etiologic exposure [1]. If a metabolite is modeled as an exposure, nondifferential measurement error can be expected to attenuate the risk association due to regression dilution bias, and if it is modeled as a confounder, this may lead to residual confounding. Differential measurement error could bias the risk association in any direction and lead to incorrect conclusions from the study [1].

Dietary intake can affect blood metabolite concentrations, and marked metabolic and hormonal changes occur in the postprandial state [2–7]. Thus, the European Federation of Clinical Chemistry and Laboratory Medicine and the Latin America Confederation of Clinical Biochemistry have recommended that blood sampling should be conducted in subjects who have not eaten in the past 12 h [8]. Further, most epidemiological studies primarily utilize fasting blood samples taken at least 6 or 8 h since last dietary intake. However, healthy individuals in Western countries spend most of their awake time in the postprandial state (~ 18 h a day), and most people only enter the fasting state during an overnight sleep [9]. Thus, measuring circulating metabolites in fasting samples may not accurately measure the true exposure. For instance, recent findings suggest that a non-fasting lipid profile is superior to fasting for predicting cardiovascular risk, and several clinical guidelines and expert consensus statements now recommend non-fasting lipid testing for most clinical evaluations [10, 11]. Further, in epidemiological studies, collecting blood samples in participants 12 h fasting may be demanding for participants and is not always feasible.

As collecting blood samples in the hours following a meal is more convenient and may more accurately measure the true exposure, it is crucial to understand how specific metabolite concentrations may change during the postprandial state. If patterns in concentrations following a habitual meal could be identified, one could more precisely account for dietary intake and time since last meal in epidemiological studies. This could improve the internal and external validity of epidemiological studies utilizing metabolomic data. Thus, the main objective of the present study was to provide a descriptive overview of metabolite and biomarker concentrations in blood in the hours after dietary intake in community-dwelling middle-aged and elderly individuals from the Hordaland Health Study. This research question has previously been explored regarding homocysteine concentrations in the same cohort [12], but herein we aim to provide a more comprehensive overview and explore amino acids, lipids, metabolites related to the one-carbon metabolism, and biomarkers of vitamin status.

## Methods

### Study population

The study population included participants from the observational community-based Hordaland Health Study, where the baseline measurements were conducted during 1997–99 in Bergen, Norway. The cohort consisted of individuals aged 46–49 years (born 1950–51, referred to as the “middle-aged group,” *n* = 3089), and individuals aged 70–74 years at baseline (born in 1925–27, referred to as the “elderly group,” *n* = 2969) who were living in the city of Bergen or neighboring suburban municipalities. The data collection was conducted as a collaboration between the National Health Screening Service (now the Norwegian Institute of Public Health), the University of Bergen, and local health services. The study design and methodology have been described in more detail elsewhere [13].

**Fig. 1:**
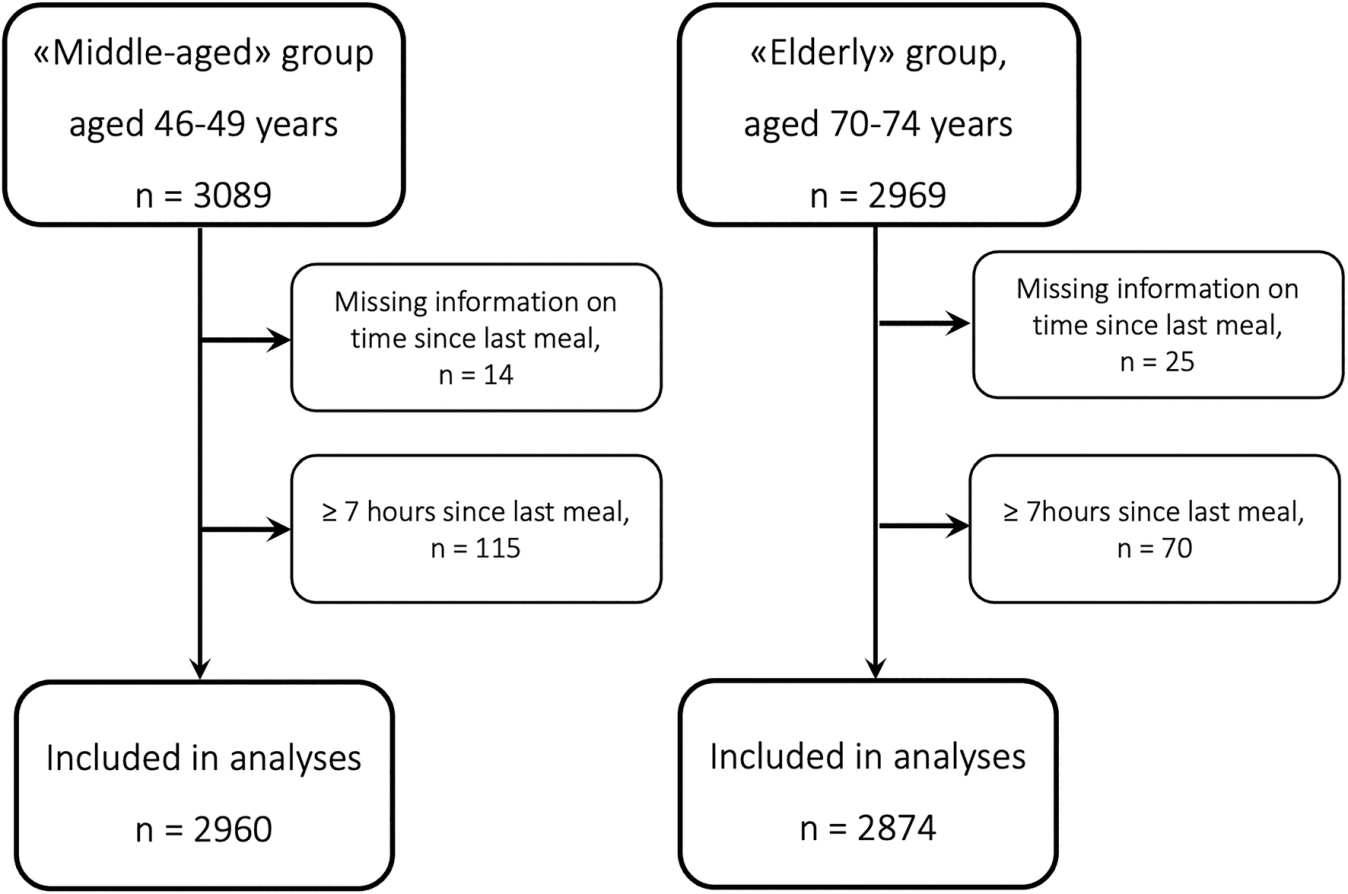
Flowchart illustrating the inclusion and exclusion process of participants in the two age cohorts in the Hordaland Health Study 1997–1999

A flowchart illustrating the inclusion and exclusion process of participants is shown in Fig. 1**.** In short, we excluded participants with missing information on time since last meal and participants with ≥ 7 h since last meal. This left us with a total of 5834 participants: 2960 participants in the middle-aged group and 2874 participants in the elderly group.

### Data collection

The collection of demographics, clinical, and biochemical characteristics has been described in more detail elsewhere [13, 14]. In short, sociodemographic data were obtained by self-administered questionnaires. Participants underwent brief health examinations including measurements of height, weight, waist and hip circumferences, and blood pressure. Hypertension was defined as the use of medication for hypertension or the mean of at least two consecutive measurements of systolic blood pressure ≥ 140 mm Hg or diastolic blood pressure ≥ 90 mm Hg. Participants were classified as diabetic based on self-reported questionnaires (previous or current diabetes), as well as a blood glucose ≥ 11.1 mmol/L within 2 h after dietary intake, or blood glucose ≥ 7 mmol/L more than 2 h after dietary intake. Information on nicotine exposure was collected by self-reported questionnaires and was verified by plasma cotinine (self-reported non-smokers with plasma cotinine levels > 85 nmol/L were classified as smokers) [15]. 

### Blood sampling and biochemical analyses

Blood samples were collected at the first visit (between 8 a.m. and 6 p.m.), and the number of hours since last meal before blood sampling was recorded. Blood samples were only collected once from each participant, and all participants attended the first visit and provided blood samples with a different number of hours since last meal. There were a low number of participants who reported that their last meal was more than 7 h previously, so we only included participants with < 7 h after the meal. The time categories were given as follows: (1) 0– < 1 h, (2) 1– < 2 h, (3) 2– < 3 h, (4) 3– < 4 h, (5) 4– < 5 h, (6) 5– < 6 h, and (7) 6– < 7 h after a meal.

Serum was obtained by collecting blood into Vacutainer tubes with no additive. Blood was allowed to clot at room temperature for 30 min before isolation of the serum fraction. Plasma samples were collected into evacuated tubes containing EDTA, chilled at 4–5 °C within 15–30 min, and then centrifuged at 4000×*g* at 10 °C for 10 min within 1–3 h. Aliquots of serum and plasma were stored at − 80 °C until analysis. Serum samples of total cholesterol, high-density lipoprotein (HDL) cholesterol, glucose, and triglycerides were analyzed within 7 days at the Department of Clinical Chemistry, Oslo University Hospital, Ullevål, with reagents from Boehringer Mannheim (Roche) as adapted to a Hitachi 911 analyzer. Cholesterol and triglycerides were measured by enzymatic methods, while HDL cholesterol was measured by a direct, enzymatic inhibition method. Low-density lipoprotein (LDL) cholesterol was calculated using the Martin/Hopkins equation as described by Martin et al. [16]. This method has been shown to have better accuracy than the Friedewald equation for estimating LDL cholesterol and is the preferred method for estimating LDL cholesterol [17]. Non-HDL cholesterol was calculated as total cholesterol minus HDL cholesterol. Analyses of amino acids, one-carbon metabolites, and B-vitamin biomarkers were conducted at the Bevital A/S Laboratory, Bergen, Norway (http://bevital.no/). All amino acids, including the one-carbon metabolites cystathionine, cysteine, glycine, homocysteine, methionine, and serine, in addition to methylmalonic acid (MMA) were measured in plasma using gas chromatography–tandem mass spectrometry [18]. Plasma betaine, choline, and dimethylglycine, as well as the B-vitamin biomarkers thiamine, thiamine monophosphate (TMP), riboflavin, flavin mononucleotide (FMN), nicotinamide, methyl-nicotinamide, pyridoxal, pyridoxal-5´-phosphate (PLP), and 4-pyridoxic acid were measured using liquid chromatography–tandem mass spectrometry (LC–MS/MS) [19, 20]. Plasma folate and cobalamin were measured by a microbiological assay [21, 22], while the lipid-soluble vitamins retinol, 25-hydroxyvitamin D, α-tocopherol, and phylloquinone were measured in plasma using LC–MS/MS [18]. More comprehensive information on the analytical platforms that were used to analyze the amino acids, one-carbon metabolites, and vitamin biomarkers is provided in Supplementary Table 1.

### Statistical analyses and presentation of the results

All statistical analyses were performed using R, version 1.4.1717 (The R Foundation for Statistical Computing, Vienna, Austria), including the packages within the *tidyverse (tidyr, dplyr, broom,* and *ggplot2)* [23], *ggtext*, and *emmeans*. Continuous variables are reported as geometric means (95% prediction intervals, PI) and categorical variables as counts (percentages). The marginal geometric mean (95% geometric CI) concentration was estimated for each time category from a linear regression model adjusted for sex, age group, and BMI and presented visually as a function of time since last meal. The *p*-value for time since last meal is presented on the figures. Unadjusted geometric mean metabolite concentrations with 95% geometric CIs at each timepoint were also plotted as a function of time since last meal for the two age cohorts and for males and females separately. To explore the potential effects of sex and age, product terms for time*sex, and time*age groups, were included in the model, and the *p*-values added to the figures.

### Ethics

The Hordaland Health Study was carried out in accordance with the Declaration of Helsinki and was approved by the Regional Committee for Medical and Health Research Ethics (REK, REK No. 2009/825) and the Norwegian Data Inspectorate. All participants provided written informed consent. The analyses presented here were approved by REK (REK No. 184165).

## Results

### Characteristics of the study participants

A total of 5834 participants were included in the statistical analyses. The main characteristics of the study participants are presented in Table 1. The middle-aged cohort (aged 46–49 years, *n* = 2960) consisted of 42.5% male participants, while the elderly cohort (aged 70–74 years, *n* = 2874) consisted of 44.6% male participants. The average BMI was slightly lower in the middle-aged cohort (25.1 kg/m^2^) compared to the elderly cohort (25.8 kg/m^2^). A total of 279 participants (9.4%) had hypertension in the middle-aged cohort, of which 130 participants were classified as hypertensive based on the use of blood pressure medications, while 149 participants were classified based on blood pressure measurements. Further, 39 participants (1.3%) were classified as diabetic, of which 23 participants were previously diagnosed with diabetes, while 16 participants were classified as having diabetes based on blood glucose levels observed during initial study sampling. In the elderly cohort, 1023 participants (35.6%) had hypertension, of which 805 used blood pressure medications. Further, 252 participants (8.8%) were diabetic, of which 184 participants had an existing diagnosis of diabetes.

**Table 1:** Main characteristics of the 5834 study participants in the Hordaland Health Study 1997–1999

	Middle-aged group (46–49 years), *n* = 2960	Elderly group (70–74 years), *n* = 2874
Age, years	47 (46, 49)	72 (70, 74)
Male, *n* (%)	1258 (42.5%)	1283 (44.6%)
Waist circumference, cm	84.7 (64.7, 111)	88.4 (67.7, 116)
Hip circumference, cm	101 (88.0, 115)	100 (87.0, 116)
Body mass index, kg/m^2^	25.1 (18.9, 33.3)	25.8 (19.2, 34.6)
Current smokers, *n* (%)	1071 (36.2%)	513 (17.8%)
Former smokers, *n* (%)	782 (26.4%)	1086 (37.8%)
Diabetes mellitus (type 1 or 2), *n* (%)	39 (1.3%)	252 (8.8%)
Hypertension, *n* (%)	279 (9.4%)	1023 (35.6%)

Continuous variables are presented as geometric means (95% prediction interval) and categorical variables as counts (%)

### Metabolite concentrations as a function of time since last meal

The estimated marginal geometric mean metabolite concentrations in the seven different time categories are provided in Table 2. Results for the two age cohorts are presented in Supplementary Table 2, and results for males and females separately are presented in Supplementary Table 3. The number of missing values of the metabolite concentrations at each timepoint is provided in Supplementary Table 4 (total cohort) and Supplementary Table 5 (missing values for the two age groups, and males and females separately).

**Table 2:** Estimated marginal geometric mean metabolite concentrations during the first 7 h after dietary intake in 5834 participants in the Hordaland Health Study 1997–1999

Hours since last meal	0– < 1	1– < 2	2– < 3	3– < 4	4– < 5	5– < 6	6– < 7
***n* participants**^**a**^	537	1628	1533	1121	701	234	81
**Serum glucose**	5.99 (5.89, 6.09)	5.46 (5.41, 55.1)	5.28 (5.23, 5.33)	5.03 (4.97, 5.09)	4.99 (4.92, 5.06)	5.06 (4.94, 5.19)	5.01 (4.81, 5.23)
**Amino acids**							
Plasma alanine, µmol/L	404 (396, 411)	422 (417, 426)	393 (389, 397)	359 (355, 364)	347 (341, 352)	333 (324, 342)	332 (317, 349)
Plasma arginine, µmol/L	48.2 (47.2, 49.3)	50.5 (49.9, 51.2)	48.3 (47.7, 48.9)	44.9 (44.3, 45.6)	43.9 (43.1, 44.7)	41.6 (40.3, 43.0)	42.6 (40.3, 45.0)
Plasma asparagine, µmol/L	50.2 (49.3, 51.0)	50.7 (50.2, 51.2)	47.8 (47.3, 48.3)	44.7 (44.1, 45.2)	44.5 (43.8, 45.2)	43.2 (42.1, 44.4)	42.5 (40.6, 44.4)
Plasma aspartic acid, µmol/L	8.66 (8.47, 8.86)	8.93 (8.82, 9.05)	8.64 (8.52, 8.76)	8.15 (8.03, 8.28)	7.84 (7.69, 8.00)	7.80 (7.54, 8.07)	7.81 (7.37, 8.27)
Plasma glutamic acid, µmol/L	95.0 (92.1, 97.9)	93.7 (92.1, 95.3)	92.8 (91.2, 94.5)	90.0 (81.1, 91.9)	89.0 (86.7, 91.4)	89.9 (85.9, 94.0)	91.9 (85.0, 99.3)
Plasma glutamine, µmol/L	522 (514, 530)	527 (522, 531)	517 (513, 522)	506 (501, 512)	516 (510, 523)	512 (501, 523)	513 (494, 532)
Plasma histidine, µmol/L	82.5 (81.6, 83.4)	83.4 (82.8, 83.9)	80.8 (80.3, 81.4)	77.3 (76.7, 77.9)	77.9 (77.2, 78.7)	75.4 (74.2, 76.7)	74.8 (72.8, 76.9)
Plasma isoleucine, µmol/L	77.5 (75.7, 79.4)	77.9 (76.8, 78.9)	73.1 (72.1, 74.1)	66.5 (65.5, 67.6)	66.9 (65.5, 68.2)	65.3 (63.1, 67.7)	62.2 (58.6, 66.1)
Plasma leucine, µmol/L	139 (136, 142)	140 (138, 142)	132 (130, 134)	122 (120, 123)	122 (120, 124)	120 (116, 123)	116 (110, 123)
Plasma lysine, µmol/L	183 (180, 186)	191 (189, 193)	183 (181, 185)	171 (169, 173)	168 (166, 171)	160 (156, 164)	156 (149, 163)
Plasma phenylalanine, µmol/L	65.4 (64.5, 66.4)	66.5 (66.0, 67.1)	63.2 (62.7, 63.8)	58.6 (58.0, 59.2)	58.0 (57.2, 58.7)	56.1 (54.9, 57.3)	57.8 (55.7, 60.0)
Plasma proline, µmol/L	220 (215, 225)	226 (223, 229)	216 (214, 219)	198 (195, 201)	190 (186, 194)	181 (175, 187)	182 (172, 193)
Plasma threonine, µmol/L	127 (125, 130)	132 (130, 133)	126 (125, 128)	119 (118, 121)	119 (117, 121)	116 (112, 119)	116 (111, 123)
Plasma tryptophan, µmol/L	69.3 (68.1, 70.5)	71.7 (71.0, 72.4)	68.6 (67.9, 69.3)	63.4 (62.7, 64.2)	61.9 (61.0, 62.8)	60.0 (58.5, 61.6)	60.5 (57.9, 63.2)
Plasma tyrosine, µmol/L	67.8 (66.4, 69.2)	70.3 (69.5, 71.1)	66.9 (66.1, 67.7)	63.3 (62.4, 64.2)	60.7 (57.9, 61.8)	59.5 (57.8, 61.4)	57.9 (55.0, 61.0)
Plasma valine, µmol/L	263 (259, 267)	265 (263, 268)	259 (256, 261)	244 (241, 247)	245 (241, 248)	238 (233, 244)	232 (222, 242)
**Lipids**							
Serum total cholesterol, mmol/L	5.78 (5.69, 5.87)	5.84 (5.78, 5.89)	5.88 (5.83, 5.94)	5.93 (5.86, 5.99	5.92 (5.84, 6.00)	5.88 (5.75, 6.02)	5.96 (5.73, 6.20)
Serum LDL cholesterol, mmol/L	3.70 (3.62, 3.78)	3.74 (3.69, 3.78)	3.80 (3.75, 3.84)	3.84 (3.78, 3.89)	3.83 (3.76, 3.90)	3.81 (3.70, 3.93)	3.95 (3.74, 4.16)
Serum HDL cholesterol, mmol/L	1.25 (1.22, 1.28)	1.27 (1.26, 1.29)	1.26 (1.25, 1.28)	1.30 (1.28, 1.32)	1.30 (1.27, 1.32)	1.27 (1.23, 1.31)	1.25 (1.19, 1.32)
Serum triglycerides, mmol/L	1.64 (1.58, 1.70)	1.61 (1.58, 1.65)	1.57 (1.54, 1.61)	1.55 (1.51, 1.59)	1.48 (1.43, 1.53)	1.48 (1.40, 1.57)	1.43 (1.29, 1.58)
**One-carbon metabolites**							
Plasma betaine, µmol/L	38.8 (37.9, 39.7)	39.6 (39.0, 40.1)	39.5 (39.0, 40.1)	37.5 (36.9, 38.1)	37.1 (36.4, 37.9)	34.9 (33.7, 36.1)	36.3 (34.2, 38.5)
Plasma choline, µmol/L	9.99 (9.81, 10.2)	10.1 (10.0, 10.2)	9.75 (9.65, 9.90)	9.58 (9.46, 9.70)	9.22 (9.07, 9.40)	8.76 (8.53, 9.0)	8.86 (8.46, 9.30)
Plasma cystathionine, µmol/L	0.22 (0.21, 0.23)	0.23 (0.23, 0.24)	0.24 (0.23, 0.24)	0.22 (0.21, 0.23)	0.22 (0.21, 0.23)	0.19 (0.18, 0.20)	0.19 (0.17, 0.21)
Plasma cysteine, µmol/L	303 (299, 307)	298 (296, 300)	302 (299, 304)	303 (300, 306)	308 (304, 311)	313 (307, 320)	316 (305, 327)
Plasma dimethylglycine, µmol/L	4.48 (4.38, 4.59)	4.60 (4.54, 4.66)	4.52 (4.46, 4.58)	4.45 (4.38, 4.52)	4.39 (4.30, 4.48)	4.24 (4.09, 4.38)	4.38 (4.14, 4.65)
Plasma glycine, µmol/L	250 (245, 256)	254 (251, 257)	251 (248, 254)	245 (242, 249)	239 (234, 243)	242 (234, 250)	236 (224, 250)
Plasma homocysteine, µmol/L	10.8 (10.5, 11.0)	10.9 (10.7, 11.0)	11.1 (10.9, 11.2)	11.0 (10.8, 11.1)	11.4 (11.2, 11.6)	11.6 (11.2, 12.1)	12.2 (11.5, 13.0)
Plasma methionine, µmol/L	24.8 (24.2, 25.4)	25.8 (25.4, 26.2)	23.8 (23.5, 24.2)	21.3 (21.0, 21.7)	20.9 (20.5, 21.4)	20.0 (19.2, 20.7)	20.2 (19.0, 21.6)
Plasma serine, µmol/L	117 (115, 119)	118 (117, 119)	114 (113, 115)	109 (108, 110)	109 (108, 111)	110 (107, 113)	108 (104, 113)
**Lipid-soluble vitamins**							
Plasma retinol, µmol/L	2.11 (2.07, 2.15)	2.16 (2.14, 2.19)	2.18 (2.15, 2.20)	2.19 (2.16, 2.22)	2.17 (2.14, 2.21)	2.14 (2.08, 2.20)	2.19 (2.08, 2.29)
Plasma 25-OH vitD, nmol/L	63.5 (61.9, 65.1)	64.6 (63.7, 65.6)	64.6 (63.7, 65.6)	65.5 (64.4, 66.7)	65.8 (64.3, 67.3)	63.5 (61.6, 66.0)	65.5 (61.3, 69.9)
Plasma α-tocopherol, µmol/L	36.2 (35.4, 37.0)	35.8 (35.4, 36.2)	36.0 (35.5, 36.4)	36.8 (36.3, 37.4)	36.3 (35.6, 37.0)	37.1 (35.9, 38.3)	36.7 (34.7, 38.7)
Plasma phylloquinone, nmol/L	1.71 (1.64, 1.80)	1.69 (1.64, 1.73)	1.62 (1.57, 1.66)	1.51 (1.47, 1.56)	1.53 (1.46, 1.58)	1.50 (1.39, 1.61)	1.28 (1.13, 1.45)
**B-vitamin biomarkers**							
Plasma thiamine, nmol/L	3.34 (3.16, 3.52)	3.42 (3.31, 3.53)	3.18 (3.08, 3.28)	3.03 (2.92, 3.15)	2.86 (2.72, 3.00)	2.65 (2.44, 2.87)	2.60 (2.34, 3.09)
Plasma TMP, nmol/L	7.52 (7.27, 7.79)	7.69 (7.54, 7.85)	7.23 (7.08, 7.38)	6.84 (6.68, 7.01)	6.90 (6.69, 7.11)	6.58 (6.24, 6.93)	6.34 (5.80, 6.93)
Plasma riboflavin, nmol/L	15.2 (14.2, 16.3)	15.0 (14.4, 15.6)	14.3 (13.7, 14.8)	14.2 (13.6, 14.9)	14.2 (13.4, 15.1)	14.4 (13.1, 16.0)	16.2 (13.7, 19.2)
Plasma FMN, nmol/L	12.6 (12.2, 13.1)	12.1 (11.8, 12.3)	12.4 (12.2, 12.7)	13.5 (13.2, 13.8)	14.4 (14.0, 14.8)	14.9 (14.2, 15.7)	15.2 (13.9, 16.6)
Plasma nicotinamide, nmol/L	367 (352, 382)	383 (375, 392)	388 (379, 397)	400 (388, 411)	374 (361, 388)	409 (385, 435)	401 (362, 445)
Plasma methyl-nicotinamide, nmol/L	85.8 (81.6, 90.2)	94.0 (91.3, 96.8)	87.6 (85.0, 90.3)	90.0 (86.9, 93.3)	85.2 (81.5, 89.0)	86.7 (80.3, 93.6)	87.3 (76.7, 99.3)
Plasma pyridoxal, nmol/L	14.1 (13.4, 14.8)	14.1 (13.7, 14.6)	13.2 (12.8, 13.6)	12.8 (12.4, 13.3)	12.6 (12.1, 13.2)	12.7 (11.7, 13.7)	13.2 (11.6, 15.0)
Plasma PLP, nmol/L	54.8 (52.2, 57.7)	56.3 (54.7, 57.9)	53.6 (52.0, 55.2)	51.8 (50.1, 53.7)	51.3 (49.1, 53.6)	51.1 (47.4, 55.1)	51.0 (44.9, 58.0)
Plasma 4-pyridoxic acid, nmol/L	27.5 (25.9, 29.1)	27.6 (26.7, 28.5)	26.5 (25.6, 27.5)	27.2 (26.1, 28.3)	26.4 (25.1, 27.8)	25.8 (23.6, 28.2)	25.8 (22.2, 30.0)
Plasma folate, nmol/L	7.13 (6.82, 7.46)	7.03 (6.85, 7.22)	6.86 (6.68, 7.05)	7.00 (6.78, 7.23)	6.91 (6.64, 7.19)	6.99 (6.53, 7.48)	6.52 (5.80, 7.32)
Plasma cobalamin, pmol/L	352 (341, 3.64)	352 (346, 359)	351 (344, 357)	355 (347, 363)	352 (342, 362)	370 (353, 389)	344 (316, 374)
Plasma MMA, µmol/L	0.20 (0.19, 0.20)	0.20 (0.20, 0.20)	0.19 (0.19, 0.20)	0.19 (0.19, 0.19)	0.19 (0.18, 0.19)	0.18 (0.18, 0.19)	0.20 (0.18, 0.21)

All values are presented as estimated marginal geometric means (95% confidence intervals), adjusted for age cohort, sex, and body mass index

*FMN* flavin mononucleotide, *HDL* high-density lipoprotein, *LDL* low-density lipoprotein, *MMA* methylmalonic acid, *PLP* pyridoxal 5’-phosphate, *TMP* thiamine monophosphate

^a^An overview of missing observations at each timepoint for each of the metabolites is found in Supplementary Table 4

### Glucose

**Fig. 2:**
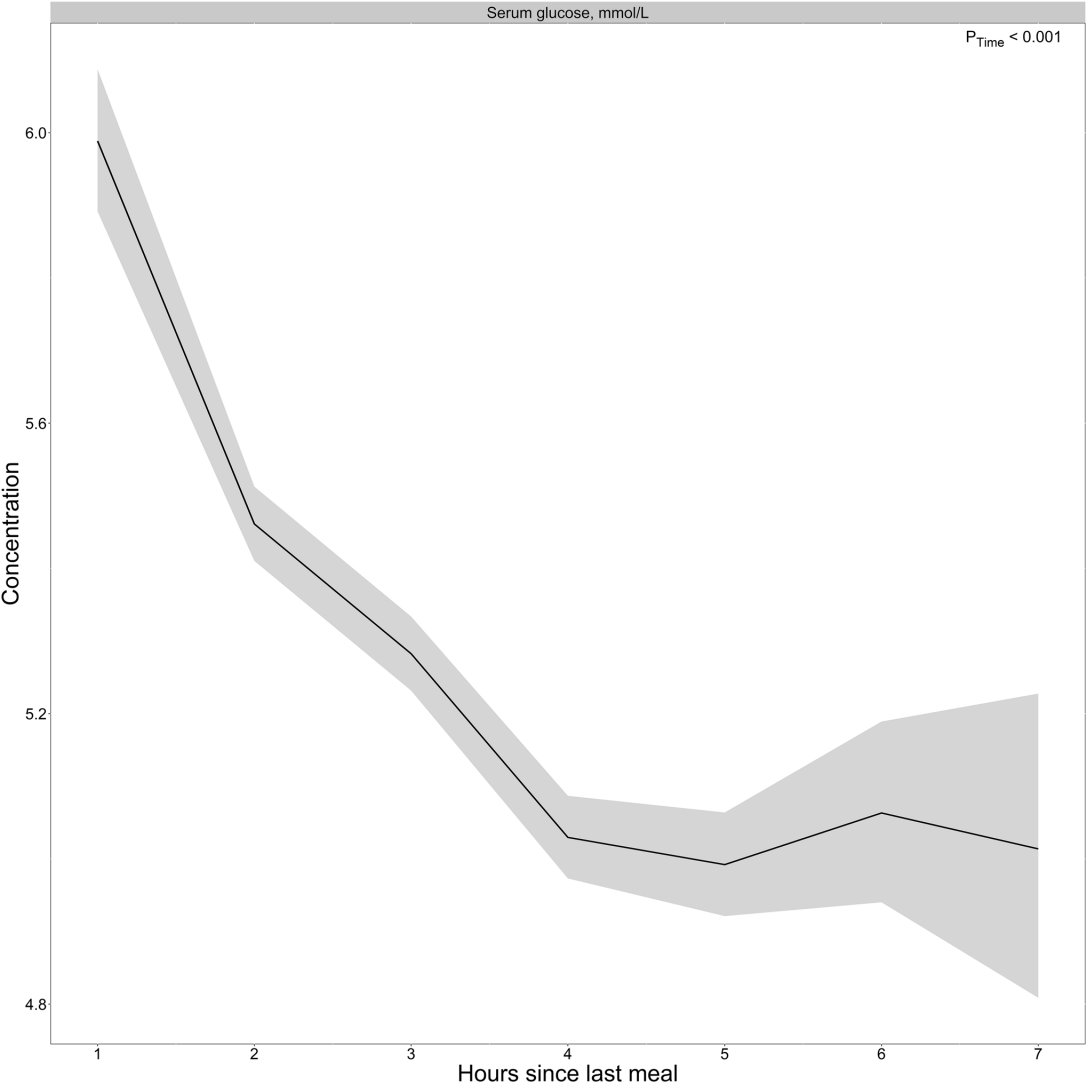
The concentration of glucose as a function of time since last meal using cross-sectional data from 5834 participants in the Hordaland Health Study 1997–1999. The solid line indicates estimated marginal geometric means (from a linear regression model adjusted for age cohort, sex, and BMI), while the shaded area represents 95% geometric confidence intervals. The *p*-value indicated in the figure is for time since last dietary intake. Note, the origin of the *y*-axis ≠ 0. An overview of the number of observations at each timepoint, and the number of missing observations for each metabolite at each timepoint is provided in Supplementary Table 4

The results for glucose concentrations as a function of time since last meal are presented in Fig. 2 and Table 2**.** As expected, we observed the highest concentrations of glucose during the 1st hour (5.99 mmol/L), with concentrations decreasing and reaching the lowest values at 4–5 h after the meal (4.99 mmol/L) and thereafter stabilizing. We observed no considerable differences in the two age groups (Supplementary Fig. 1) or between sexes (Supplementary Fig. 2).

### Amino acids

**Fig. 3:**
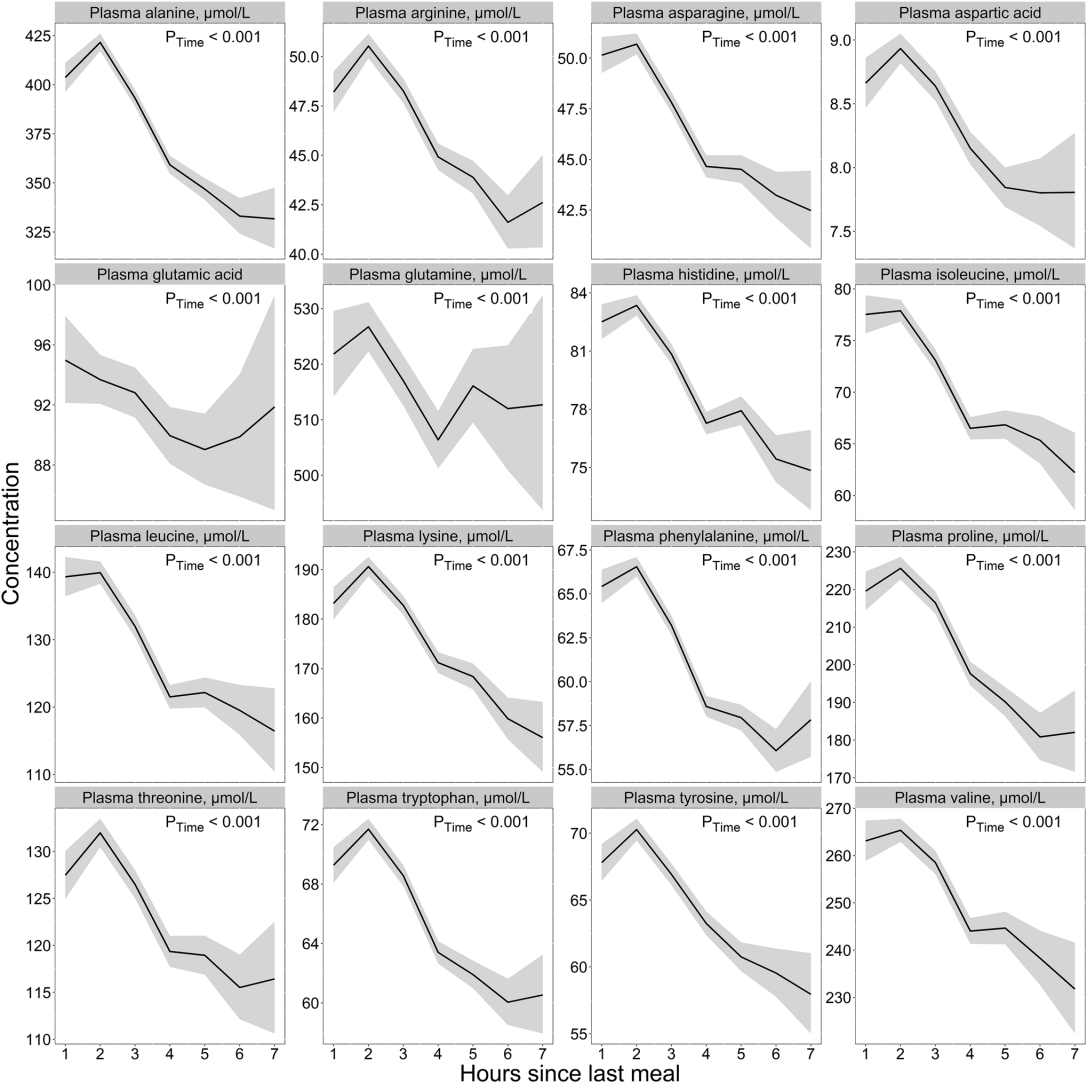
The concentration of amino acids as a function of time since last meal using cross-sectional data from 5834 participants in the Hordaland Health Study 1997–1999. The solid line indicates estimated marginal geometric means (from a linear regression model adjusted for age cohort, sex, and BMI), while the shaded area represents 95% geometric confidence intervals. The *p*-value indicated in the figure is for time since last dietary intake. Note, the origin of the *y*-axis ≠ 0, and the *y*-axes are scaled to be compatible with the metabolite concentration ranges. An overview of the number of observations at each timepoint, and the number of missing observations for each metabolite at each timepoint is provided in Supplementary Table 4

Among the amino acids, we observed a common pattern for alanine, arginine, asparagine, aspartic acid, histidine, isoleucine, leucine, lysine, phenylalanine, proline, threonine, tryptophan, tyrosine, and valine **(**Fig. 3**, **Table 2**)**. The concentrations of these amino acids were highest 1–2 h after a meal, with the lowest concentrations observed at 5–7 h. The difference between the highest and the lowest values was ≥ 10% for all these amino acids, with the largest differences found for alanine (90 µmol/L, 27%), isoleucine (15.7 µmol/L, 25.2%), and proline (45 µmol/L, 24.9%). Findings were consistent in both age cohorts (Supplementary Fig. 3) and both sexes (Supplementary Fig. 4). No consistent patterns were observed for glutamic acid or glutamine.

### Lipids

**Fig. 4:**
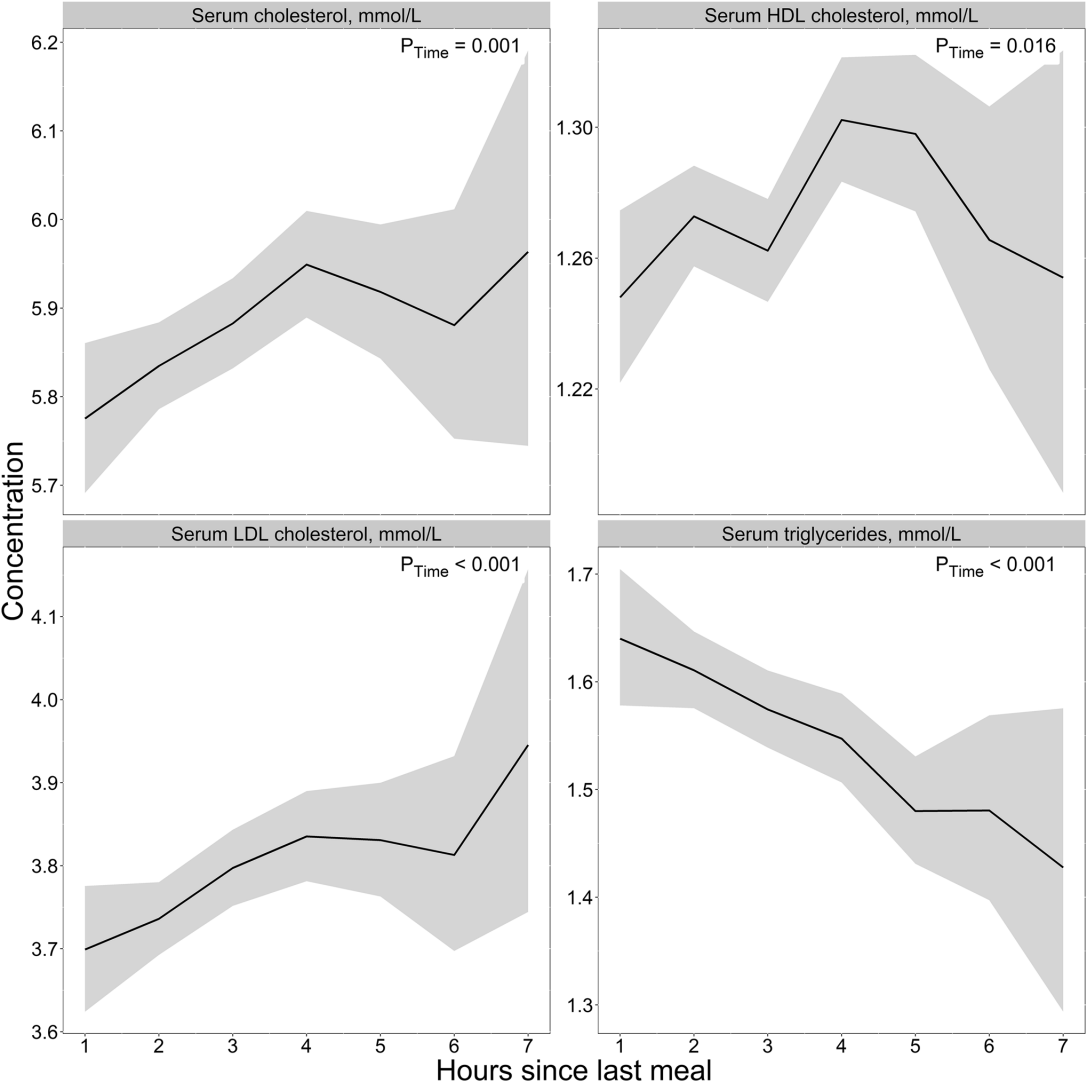
The concentration of blood lipids as a function of time since last meal using cross-sectional data from 5834 participants in the Hordaland Health Study 1997–1999. The solid line indicates estimated marginal geometric means (from a linear regression model adjusted for age cohort, sex, and BMI), while the shaded area represents 95% geometric confidence intervals. The *p*-value indicated in the figure is for time since last dietary intake. Note, the origin of the *y*-axis ≠ 0, and the *y*-axes are scaled to be compatible with the metabolite concentration ranges. An overview of the number of observations at each timepoint, and the number of missing observations for each metabolite at each timepoint is provided in Supplementary Table 4. *HDL* high-density lipoprotein, *LDL* low-density lipoprotein

For the lipids **(**Fig. 4**, **Table 2**),** we observed that the concentrations of total cholesterol and LDL cholesterol in the total cohort were lowest in the first 2 h after a meal (5.78 and 3.70 mmol/L, respectively) and highest 6–7 h after a meal (5.96 and 3.95 mmol/L). The maximum mean difference between the lowest and the highest values was 0.18 mmol/L (3.1%) for total cholesterol and 0.25 mmol/L (6.8%) for LDL cholesterol. We observed some age differences, with concentrations being highest at 6–7 h after a meal in the middle-aged group, while in the elderly group, the concentrations were highest at 3–5 h after a meal (Supplementary Fig. 5). Further, HDL cholesterol concentrations were highest at 4–5 h after a meal (1.30 mmol/L) and lowest at 1–2 and 6–7 h after food intake (1.25 mmol/L). For the triglycerides, we observed the highest concentrations the first 2 h after a meal (1.64 mmol/L) and lower concentrations thereafter (2–7 h), with the lowest concentrations observed at 6–7 h after a meal (1.43 mmol/L), a difference of 0.21 mmol/L (14.7%). We observed no considerable sex or age differences for HDL or the triglycerides (Supplementary Fig. 5 and Supplementary Fig. 6).

### One-carbon metabolites

**Fig. 5:**
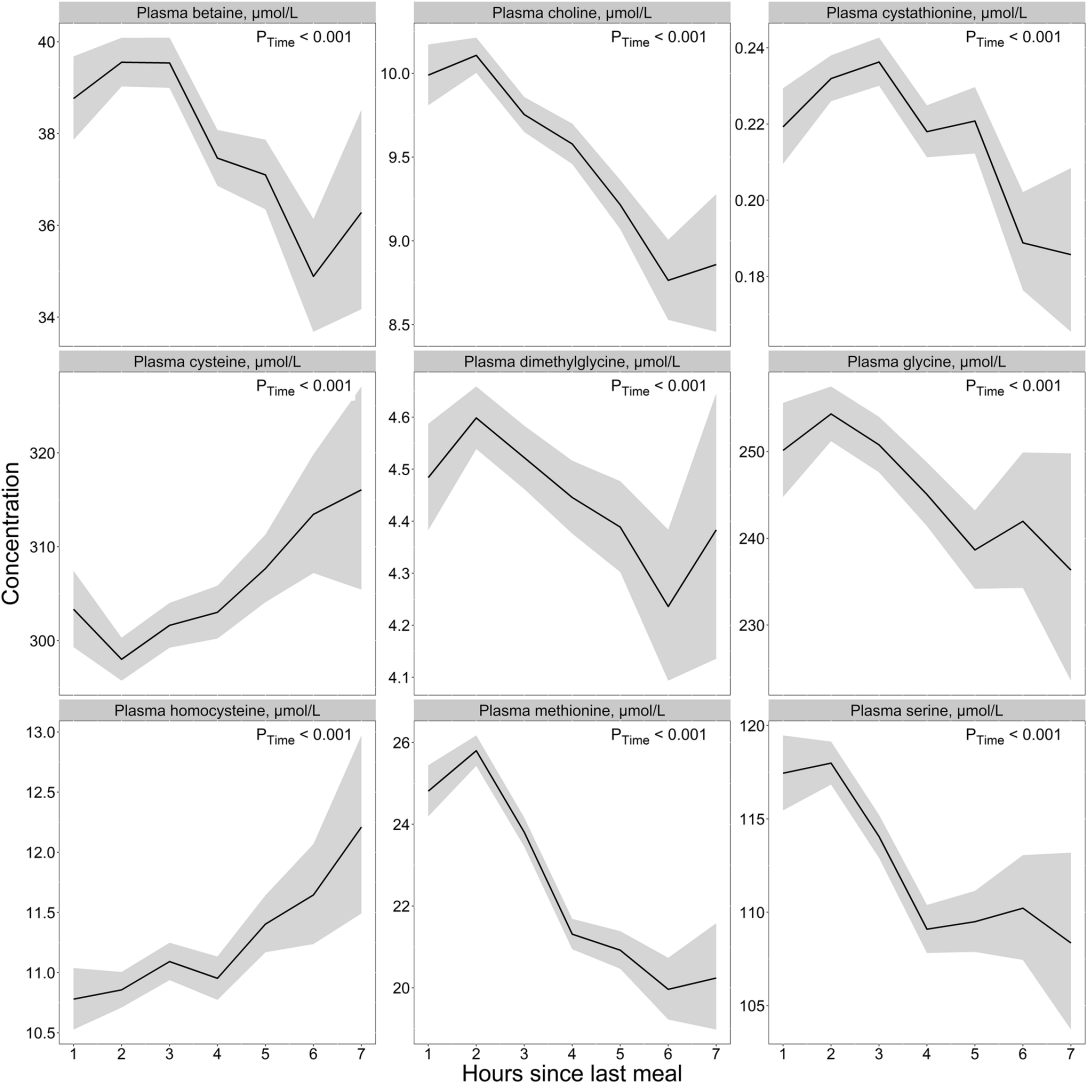
The concentration of one-carbon metabolites as a function of time since last meal using cross-sectional data from 5834 participants in the Hordaland Health Study 1997–1999. The solid line indicates estimated marginal geometric means (from a linear regression model adjusted for age cohort, sex, and BMI), while the shaded area represents 95% geometric confidence intervals. The *p*-value indicated in the figure is for time since last dietary intake. Note, the origin of the *y*-axis ≠ 0, and the *y*-axes are scaled to be compatible with the metabolite concentration ranges. An overview of the number of observations at each timepoint, and the number of missing observations for each metabolite at each timepoint is provided in Supplementary Table 4

For the one-carbon metabolites **(**Fig. 5**, **Table 2*),* we observed that the levels of homocysteine and cysteine were lowest during the 1st hours after a meal (10.8 µmol/L and 298 µmol/L, respectively), with concentrations peaking at 6–7 h (12.2 µmol/L and 316 µmol/L). For betaine, choline, cystathionine, dimethylglycine, glycine, methionine, and serine, we observed a pattern with the highest concentrations 1–2 h after a meal, and the lowest concentrations usually observed at 5–7 h after a meal. The relative difference between the highest and the lowest values was lowest for cysteine (18 µmol/L, 6.0%), glycine (18 µmol/L, 7.6%), and dimethylglycine (0.36 µmol/L, 8.5%) and highest for methionine (5.8 µmol/L, 29.0%) and cystathionine (0.05 µmol/L, 26.3%). We observed no noteworthy age or sex differences in the concentrations of any of the one-carbon metabolites as a function of time since last meal (Supplementary Figs. 7 and 8, respectively).

### Lipid-soluble vitamins

**Fig. 6:**
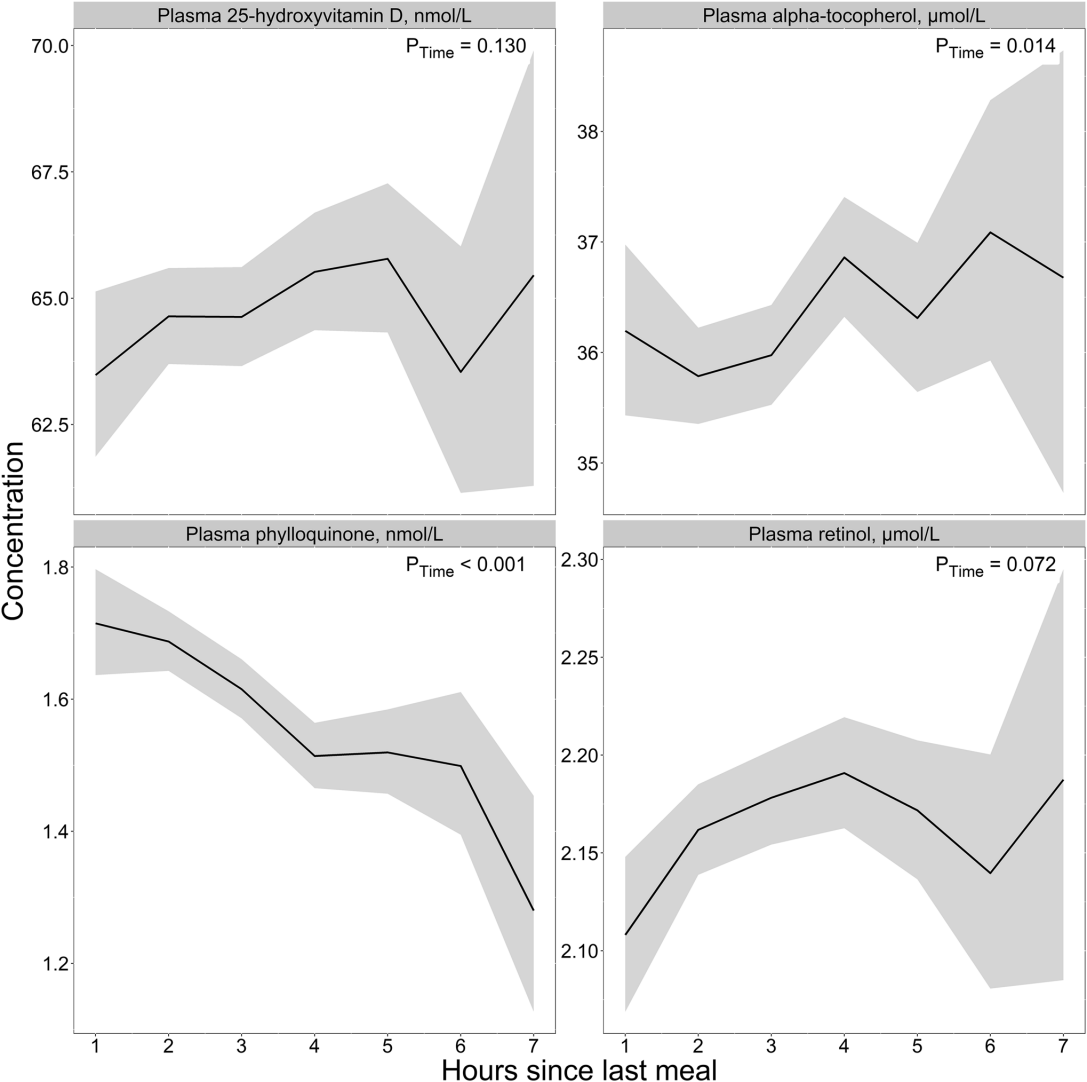
The concentration of lipid-soluble vitamins as a function of time since last meal using cross-sectional data from 5834 participants in the Hordaland Health Study 1997–1999. The solid line indicates estimated marginal geometric means (from a linear regression model adjusted for age cohort, sex, and BMI), while the shaded area represents 95% geometric confidence intervals. The *p*-value indicated in the figure is for time since last dietary intake. Note, the origin of the *y*-axis ≠ 0, and the *y*-axes are scaled to be compatible with the metabolite concentration ranges. An overview of the number of observations at each timepoint, and the number of missing observations for each metabolite at each timepoint is provided in Supplementary Table 4

Among the lipid-soluble vitamins **(**Fig. 6**, **Table 2**)**, no clear patterns were observed for retinol, 25-hydroxyvitamin D, or α-tocopherol. For phylloquinone, we observed peak concentrations in the 1st hour after a meal (1.71 nmol/L) and lower concentrations thereafter (2–7 h), with the lowest values observed at 6–7 h after a meal (1.28 nmol/L), giving a maximum mean difference of 0.43 nmol/L (33.6%). These observations were also observed in both age cohorts (Supplementary Fig. 9) and both sexes (Supplementary Fig. 10).

### B-vitamin status

**Fig. 7:**
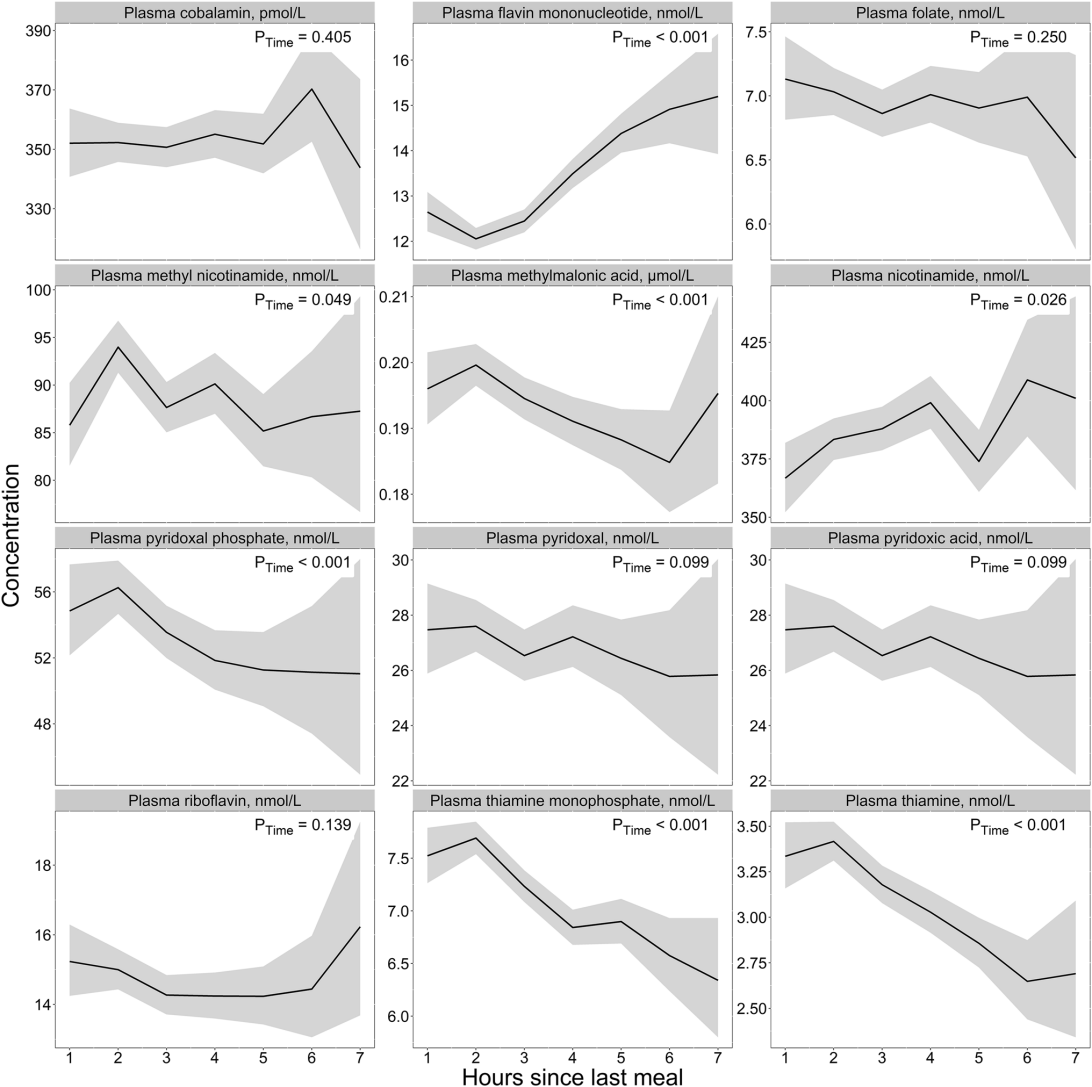
The concentration of B-vitamin biomarkers as a function of time since last meal using cross-sectional data from 5834 participants in the Hordaland Health Study 1997–1999. The solid line indicates estimated marginal geometric means (from a linear regression model adjusted for age cohort, sex, and BMI), while the shaded area represents 95% geometric confidence intervals. The *p*-value indicated in the figure is for time since last dietary intake. Note, the origin of the *y*-axis ≠ 0, and the *y*-axes are scaled to be compatible with the metabolite concentration ranges. An overview of the number of observations at each timepoint, and the number of missing observations for each metabolite at each timepoint is provided in Supplementary Table 4

Results for the B-vitamin markers are given in Fig. 7 and Table 2. We observed that the concentrations of thiamine and TMP were highest in the first 2 h after a meal (3.42 nmol/L and 7.69 nmol/L, respectively), before steadily declining to their lowest concentrations at 6–7 h (2.60 nmol/L and 6.34 nmol/L), giving a difference between the highest and the lowest values of 31.5% for thiamine and 21.3% for TMP. For FMN, the opposite was true, with the lowest concentration observed during the first 2 h after a meal (12.1 nmol/L), and higher concentrations being observed with increasing time since dietary intake, with the highest concentrations observed at 6–7 h after dietary intake (15.2 nmol/L). No clear patterns emerged for the other B-vitamin biomarkers, including riboflavin, nicotinamide, methyl nicotinamide, pyridoxal, PLP, 4-pyridoxic acid, folate, cobalamin, or MMA. Findings were consistent in both age groups (Supplementary Fig. 11) and both sexes (Supplementary Fig. 12).

## Discussion

In this study, using cross-sectional data from a large Norwegian cohort including two distinct age groups of community-dwelling adults, we investigated circulating metabolite concentrations as a function of time since last meal. For most amino acids, we observed highest concentrations during the first 3 h after a meal, which was also observed for the one-carbon metabolites betaine, choline, cystathionine, dimethylglycine, glycine, methionine, and serine. Among the lipids, we observed the lowest concentrations of total and LDL cholesterol and the highest concentrations of triglycerides the first 2 h after a meal. Lastly, we observed the lowest concentrations of FMN and the highest concentrations of thiamine, TMP, and phylloquinone during the first 2 h after the last meal.

Our findings for glucose are consistent with what is already known from the previous literature, with peak glucose concentrations ~ 1 h after the start of a meal, returning to preprandial levels within a few hours [24]. Thus, the results for glucose may be used as a validation marker for the other results. Further, our amino acid observations are consistent with findings reported elsewhere. However, most of these studies reported concentrations following the ingestion of specific foods or nutrients, for instance, dairy products [25], or comparing different types or amounts of protein [26–29]. The results from the present study indicate that circulating amino acid concentrations likely change after a habitual meal. In light of established knowledge about protein metabolism, wherein proteins are cleaved into amino acids which are transported in blood after absorption, these findings are arguably, as expected [30]. Our observations concerning the B-vitamin biomarkers are also in line with previously published reports. In an intervention study, comparing circulating B-vitamin concentrations 5 h after two different meals, Sharma et al. [31] reported that the concentrations of thiamine were the lowest right after an overnight fast, with the concentrations peaking in the first 2 h after a meal and decreasing thereafter until 5 h after a meal, which was also observed in the present study. The peak in both thiamine and TMP during the first 2 h after a meal, as observed in the present study, is likely attributable to thiamine content in the food, as both free thiamine and TMP enter the bloodstream during absorption of thiamine [32]. For FMN, Sharma et al. reported the highest concentrations immediately before a meal, decreased concentrations immediately after meal ingestion, and increased concentrations thereafter, comparable to the present findings. FMN serves as a cofactor in the electron transport chain, and the lower concentrations during the 1st hour after a meal may indicate increased utilization of FMN as a cofactor. Comparable to the present study, Sharma et al. also observed relatively stable concentrations of the Vitamin B6 vitamers (pyridoxal, PLP, and pyridoxic acid) [31]. For postprandial lipid concentrations, most studies have investigated the response to high-fat meals [33–36]. However, some studies have investigated blood lipid profiles following non-standardized meals. Using cross-sectional data from 33.391 participants in the Copenhagen General Population Study, Langsted et al. [37] reported the highest concentrations of triglycerides during the 1st hours after a meal, with a maximum mean difference of 0.3 mmol/L, which is comparable to the present findings (0.21 mmol/L). Peak concentrations directly after a meal are likely attributable to fat intake from the meal [37, 38]. Lower concentrations of total and LDL cholesterol the 1st hours after a meal, as observed in the present study, have also been reported by others [37, 39]. Langsted et al. suggested that the observed drop in concentrations could be caused by a hemodilution effect from fluid intake in relation to the meal [37]; however, it has been argued that mechanisms other than hemodilution must be involved [39, 40]. One possible explanation may be attributed to increased hydrolyzation of triglycerides in chylomicrons after a meal, catalyzed by the enzyme lipoprotein lipase (LPL), which subsequently inhibits the formation of LDL from very low-density lipoproteins (VLDLs), as VLDL and chylomicrons compete for LPL [41, 42].

It is crucial to mention that although the findings from the present study indicate that the concentrations of several metabolites change the first 7 h after a meal, these potential changes are not necessarily good, bad, or abnormal. Metabolite concentrations are not static but fluctuate during the day, *e.g.,* after dietary intake, reflecting normal biological variations. With that said, knowing how metabolite concentrations change after a meal is crucial when interpreting metabolite data. In this study, we observed a maximum mean difference of 0.18 mmol/L for total cholesterol, 0.25 mmol/L for LDL cholesterol, and 0.21 mmol/L for triglycerides. These differences are evaluated to be clinically insignificant, as stated by the joint consensus statement from the European Atherosclerosis Society and the European Federation of Clinical Chemistry and Laboratory Medicine in 2016 [10]. However, it is common in clinical practice today to use cutoffs to diagnose or initiate a treatment, and even small changes in concentrations may cause a patient to cross the given cutoff. Thus, if relying on a single measurement, a patient may risk being classified as diseased or non-diseased or given treatment depending on the time since last meal at the time of blood sampling.

Further, the information obtained from this study may be of importance in research settings, where biomarkers may be used to evaluate the effect of a supplement or a treatment, or in epidemiological studies, where metabolites may be modeled as exposures, outcomes, or confounders of associations. In these situations, even modest differences in metabolite concentrations could be of importance. For instance, we observed consistently higher values of plasma homocysteine with increasing time since last dietary intake, with a maximum mean difference of 1.07 µmol/L (13%). Homocysteine has been extensively investigated in epidemiological studies as a risk factor or a marker of a variety of diseases including cardiovascular disease [43] and dementia [44], among others. Similarly, results from epidemiological studies suggest that the branched chained amino acids (BCAAs; leucine, isoleucine, and valine) are biomarkers for increased risk of diabetes [45]. In this study, we observed a maximum difference in leucine of 24 µmol/L (20.7%), isoleucine of 15.7 µmol/L (25.2%), and valine of 33 µmol/L (14.2%). In existing epidemiological studies on the association between the BCAAs and diabetes, information on prandial status at the time of blood sampling is rarely reported [45]. Here, we argue that blood sampling at random hours after a meal may give rise to uncertainty in the results when investigating the association between several metabolites in associations with diseases. Also, when the metabolite concentration changes in the hours after food intake, and the timing of blood collection relative to food intake differs systematically across baseline risk (i.e., blood samples from higher-risk individuals being collected shorter or longer after the last meal), this could attenuate or accentuate the observed association. It is common in epidemiological studies today to distinguish between blood samples taken in the so-called “non-fasting” and “fasting” states, usually based on a cutoff at 6, 8, or 12 h since last meal. In many studies, only metabolites measured in blood samples taken more than 6 or 8 h since last dietary intake are included. However, using cutoffs to categorize an underlying continuous variable assumes homogeneity within the categories, with a sharp discontinuity at the cutoff [46]. Krüg et al. [47] have previously demonstrated that several metabolites change during prolonged fasting. Our findings indicate that many metabolite concentrations change considerably within the first 7 h after dietary intake, which would usually be classified as the “non-fasting” state, among them many metabolites regarded as risk factors for non-communicable diseases. Thus, our findings suggest that it may not be sufficient only to account for dietary intake by distinguishing between the fasting and the non-fasting state but should be done by accounting for the exact time since last meal. As stated, measuring metabolites in the postprandial state could be a better measure of true exposure when using metabolites data in epidemiological studies. When using existing epidemiological data, researchers could, if the information is available, consider adjusting for the exact number of hours since the last meal when modeling metabolites as an exposure or an outcome. This could reduce the excess variation introduced by collecting blood samples at different time since last meal. When collecting data in the future epidemiological studies, the time of blood sampling should be standardized as much as possible with regard to the time of day and time after dietary intake, and preferably after a standardized meal, to reduce external influence on metabolite concentrations. Should standardization not be possible, then accurate recording of time since last meal for all participants is imperative.

The major strength of this study is the large sample size, including nearly 6000 participants. Further, the narrow age range in the two age cohorts (46–49 and 70–74 years) is considered a strength in this study, as age may affect metabolite concentrations, and thus contributes to variability in the results. The importance of including different age groups is also supported by the observation of, on average, different concentrations according to age for some of the metabolites. The inclusion of both males and females may also be considered a strength of the present study in terms of generalizability. However, usually, there are sex differences in absolute energy and nutrient intakes, which may have driven some of the differences between males and females that we observed for some metabolites. An evident limitation of the study is the cross-sectional design with only a single blood sample from each participant which did not allow for investigation of within-individual changes in metabolite concentrations. Thus, the results must be interpreted as patterns, rather than changes in metabolite concentrations in the postprandial period. Prior to blood sampling, no preparatory instructions were given to the participants on what or when to eat. Thus, the blood samples were taken after meals of varying composition, and no information concerning what the participants ate before the blood sampling was available. The lack of a standardized meal prior to blood sampling may have introduced variability to the results [48]. However, the absence of a standardized meal in the present study could also be interpreted as a strength as it represents a “real-world” setting where blood sampling is usually conducted without any preparatory instructions to the patient or participants on what to eat. The blood sampling was conducted at different times of day and during different seasons of the year. It has been reported that some metabolites exhibit a circadian rhythm, with lipids and amino acids as the most frequently observed rhythmic metabolites [49]. It is also well-known that in populations living far from the Earth´s equator, like Norway, the population concentrations of 25-hydroxyvitamin D, tend to change during seasons [50]. However, there is no reason to believe that there are systematic differences in time of day or season of blood sampling distributed across the time since last meal categories. Thus, we may assume that these are sources of random error and give rise to natural variability in the results. Lastly, the number of observations in the different time categories varied, with a lower number of observations 5–7 h after a meal, making the results at these timepoints subject to greater uncertainty.

## Conclusion

In this study of community-dwelling Norwegian middle-aged and elderly adults, we observed patterns among most amino acids and one-carbon metabolites with peak concentrations occurring in the 1st hours after a meal. Concentrations of homocysteine and cysteine were lowest right after the meal, peaking at 6–7 h. The concentrations of phylloquinone and triglycerides were highest 1 h after dietary intake. Thiamine and TMP concentrations were highest, while the concentration of FMN was lowest within the first 2 h after a meal. No clear patterns emerged for the other fat-soluble vitamins, blood lipids, or B-vitamin biomarkers. Our findings indicate that many metabolites and biomarkers change during the first 7 h after a habitual meal, and suggest that the current practice of broadly distinguishing between fasting and non-fasting blood samples in clinical and research settings may be imprecise and inadequate. If confirmed in the future studies, this may have implications for how to account for dietary intake and time since last meal when using existing data, and for the collection of blood samples in the future studies.

## Supplementary Information

Below is the link to the electronic supplementary material.

Supplementary file1 (PDF 2547 KB)

## Data Availability

Data described in the manuscript, code book, and analytic code will be made available upon request pending application and approval.
