# Supplementary material for: Exploratory analyses on the effect of time since last meal on concentrations of amino acids, lipids, one-carbon metabolites, and vitamins in the Hordaland Health Study
Source: Eur J Nutr. 2023 Jul 27;62(7):3079–95. doi: 10.1007/s00394-023-03211-y (PMC10468919; doi:10.1007/s00394-023-03211-y)
Supplement: Supplementary file 1 — Supplementary file1 (PDF 2547 KB) [file 394_2023_3211_MOESM1_ESM.pdf]

Supplementary Material: European Journal of Nutrition

Exploratory analyses on the effect of time since last meal on patterns of amino acids, lipids, one carbon metabolites, and vitamins in the Hordaland Health Study

Authors: Åslaug Matre Anfinsen, Hanne Rosendahl-Riise, Ottar Nygård, Grethe S. Tell, Per Magne Ueland, Arve Ulvik, Adrian McCann, Jutta Dierkes, Vegard Lysne

Corresponding author:

Åslaug Matre Anfinsen

Department of Clinical Science, University of Bergen

Haukelandsbakken 15, 5009 Bergen, Norway

E-mail: aslaug.matre@uib.no

List of materials included in the Supplementary Information

Supplementary Tables

Supplementary Table 1. Main characteristics of the analytical platforms used to analyze the amino acids, one-carbon metabolites, and vitamin biomarkers in the Hordaland Health Study 1997-99
Supplementary Table 2. The geometric means of metabolite concentrations at each timepoint in middle-aged (M) and elderly (E) adults in the Hordaland Health Study 1997-1999[1]
Supplementary Table 3. The geometric means of metabolite concentrations at each timepoint in males and females in the Hordaland Health Study 1997-99
Supplementary Table 4. The number of missing values for each metabolite at each timepoint in the Hordaland Health Study 1997-99
Supplementary Table 5. The number of missing observations at each timepoint in middle-aged and elderly adults, and among males and females in the Hordaland Health Study 1997-1999

Supplementary Figures

Supplementary Figure 1. The concentration of glucose as a function of time since the last meal in the two age cohorts.
Supplementary Figure 2. The concentration of glucose as a function of time since the last meal in males and females separately.
Supplementary Figure 3. The concentration of amino acids as a function of time since the last meal in the two age cohorts.
Supplementary Figure 4. The concentration of amino acids as a function of time since the last meal in males and females separately.
Supplementary Figure 5. The concentration of blood lipids as a function of time since the last meal in the two age cohorts.
Supplementary Figure 6. The concentration of blood lipids as a function of time since the last meal in males and females separately.
Supplementary Figure 7. The concentration of one-carbon metabolites as a function of time since the last meal in the two age cohorts.
Supplementary Figure 8. The concentration of one-carbon metabolites as a function of time since the last meal in males and females separately.
Supplementary Figure 9. The concentration of lipid-soluble vitamins as a function of time since the last meal in

the two age cohorts.

Supplementary Figure 10. The concentration of lipid-soluble vitamins as a function of time since the last meal in males and females separately.

Supplementary Figure 11. The concentration of B-vitamin biomarkers as a function of time since the last meal in the two age cohorts.

Supplementary Figure 12. The concentration of B-vitamin biomarkers as a function of time since the last meal in males and females separately.

Supplementary Table 1. Main characteristics of the analytical platforms used to analyze the amino acids, one-carbon metabolites, and vitamin biomarkers in the Hordaland Health Study 1997-99[1]

Platform	GC	LC1	LC2	LC3
Type of analysis	GC-MS/MS	LC-MS/MS	LC-MS/MS	LC-MS/MS
Metabolites analyzed (year of analysis)	Alanine (2017)<br>Arginine (2017)<br>Asparagine (2017)<br>Aspartic acid (2017)<br>Glutamic acid (2017)<br>Glutamine (2017)<br>Histidine (2017)<br>Isoleucine (2017)<br>Leucine (2017)<br>Lysine (2017)<br>Phenylalanine (2017)<br>Proline (2017)<br>Threonine (2017)<br>Tryptophan (2010)<br>Tyrosine (2017)<br>Valine (2017)<br>Methyl-malonic acid (2017)<br>Cysteine (2010)<br>Cystathionine (2010)	Betaine (2010)<br>Choline (2010)<br>DMG (2010)	Thiamine (2017)<br>Thiamine monophosphate (2017)<br>Riboflavin (2010)<br>Flavin mononucleotide (2010)<br>Nicotinamide (2017)<br>Methyl nicotinamide (2017)<br>Pyridoxal (2010)<br>Pyridoxal-5´-phosphate (2010)<br>4´-pyridoxic acid (2010)	Retinol (2017)<br>25-hydroxyvitamin D (2017)<br>α-tocopherol (2017)<br>Phylloquinone (2017)

	Glycine (2017)<br>Homocysteine (2010)<br>Methionine (2010)<br>Serine (2017)			
**Pretreatment**	Dithioerythriol	-	-	-
**Protein precipitation**	Ethanol	Trichloroacetic acid	Trichloroacetic acid	Ethanol
**Liquid-liquid extraction**	Isooctane/chloroform	-	-	Isooctane/chloroform
**Derivatization**	Methylchloroformate	-	-	-
**Column**	CP Sil 24 CB	150x4.6 mm, 3 um, phenyl	150x4.6 mm, 3.5 um, C8	50x4.6 mm, 2.7 um, C18
**Mobile phase**	Helium	Acetic acid:methanol	Acetic acid:HFBA:acetonitrile	Methanol:NH_4-formate
**Elution**	Temperature step gradient	Isocratic	Step gradient	Isocratic
**MS, ion source**	Electrospray ionization, positive mode	Electrospray ionization, positive mode	Electrospray ionization, positive mode	Atmospheric chemical ionization, positive mode

[1]All analyses were conducted at Bevital AS (http://bevital.no/). **Abbreviations:** GC-MS, Gas chromatography mass spectometry; HFBA, Heptafluorobutyric acid; LC-MS, liquid chromatography mass spectometry

Supplementary Table 2. The geometric means of metabolite concentrations at each timepoint in middle-aged (M, *n* = 2960) and elderly (E, *n* = 2874) adults in the Hordaland Health Study 1997-1999[1]

**Hours after meal**		**0-<1**	**1-<2**	**2-<3**	**3-<4**	**4-<5**	**5-<6**	**6-<7**
***n* participants**	M[2]	369	916	731	514	276	113	41
	E[2]	168	712	803	606	425	121	40
**Serum glucose, mmol/L**	M	5.67	5.19	5.04	4.88	4.87	4.99	5.03
	E	6.38	5.73	5.50	5.17	5.15	5.14	5.00
**Amino acids**								
Plasma alanine, µmol/L	M	390	406	374	347	334	324	326
	E	412	434	408	367	359	342	338
Plasma arginine, µmol/L	M	47.4	50.3	47.3	43.7	43.0	40.2	43.4
	E	48.1	50.5	48.9	45.6	44.3	42.9	41.8
Plasma asparagine, µmol/L	M	51.1	51.9	48.4	45.3	45.3	44.1	43.0
	E	49.2	49.5	47.2	43.9	43.3	42.2	41.9
Plasma aspartic acid, µmol/L	M	8.84	9.06	8.75	8.27	7.95	8.35	7.70
	E	8.34	8.73	8.39	7.89	7.66	7.28	7.91
Plasma glutamic acid, µmol/L	M	88.8	87.6	87.0	85.3	82.5	84.7	82.9
	E	99.9	98.3	96.0	92.0	94.9	94.9	102
Plasma glutamine, µmol/L	M	517	520	508	496	505	496	498
	E	521	533	524	514	522	526	528
Plasma histidine, µmol/L	M	84.6	86.1	82.5	79.4	79.8	77.2	75.3
	E	80.2	80.3	78.6	74.7	75.6	73.6	74.4
Plasma isoleucine, µmol/L	M	75.7	76.0	70.1	65.3	65.9	65.5	60.6
	E	77.9	78.4	74.1	65.9	67.3	64.9	63.9
Plasma leucine, µmol/L	M	138	138	128	121	122	121	115
	E	138	139	132	119	121	117	118
Plasma lysine, µmol/L	M	179	188	176	168	164	157	150
	E	186	191	188	173	172	162	162
Plasma phenylalanine, µmol/L	M	63.3	64.6	61.3	57.4	56.6	55.2	56.6
	E	67.8	68.2	64.7	59.4	59.3	57.0	59.1
Plasma proline, µmol/L	M	212	217	210	191	181	179	169
	E	222	232	218	198	196	181	195
Plasma threonine, µmol/L	M	132	137	128	123	124	120	124
	E	122	127	124	115	114	111	109
Plasma tryptophan, µmol/L	M	70.4	73.3	69.1	64.6	62.1	61.9	63.5
	E	67.4	69.4	67.0	61.3	60.8	58.1	57.6
Plasma tyrosine, µmol/L	M	64.0	67.0	63.6	60.5	58.2	56.6	55.6

	E	72.5	73.4	70.1	66.0	63.7	62.7	60.4
Plasma valine, µmol/L	M	257	259	250	241	241	238	227
	E	266	268	262	242	248	238	237
**Blood lipis**								
Serum total cholesterol, mmol/L	M	5.57	5.61	5.63	5.68	5.66	5.68	5.79
	E	5.98	6.09	6.19	6.24	6.23	6.11	6.14
Serum LDL-cholesterol, mmol/L	M	3.53	3.54	3.57	3.66	3.62	3.65	3.83
	E	3.86	3.95	4.05	4.04	4.08	3.99	4.06
Serum HDL-cholesterol, mmol/L	M	1.25	1.29	1.27	1.28	1.28	1.22	1.25
	E	1.28	1.28	1.30	1.37	1.33	1.31	1.26
Serum triglycerides, mmol/L	M	1.54	1.49	1.46	1.48	1.40	1.44	1.29
	E	1.71	1.71	1.64	1.58	1.59	1.52	1.58
**One-carbon metabolites**								
Plasma betaine, µmol/L	M	36.4	37.5	37.4	34.4	35.5	34.2	35.9
	E	40.4	40.9	40.3	38.7	37.6	35.1	36.5
Plasma choline, µmol/L	M	9.46	9.58	9.20	9.01	8.66	8.48	8.24
	E	10.4	10.6	10.2	9.99	9.70	9.03	9.52
Plasma cysteine, µmol/L	M	288	281	285	285	295	294	293
	E	316	316	318	321	323	335	341
Plasma cystathionine, µmol/L	M	0.18	0.19	0.20	0.18	0.18	0.16	0.15
	E	0.26	0.28	0.27	0.26	0.27	0.23	0.22
Plasma dimethylglycine, µmol/L	M	4.39	4.52	4.47	4.33	4.34	4.31	4.37
	E	4.55	4.63	4.50	4.46	4.38	4.15	4.39
Plasma glycine, µmol/L	M	255	258	254	249	241	237	244
	E	248	254	254	248	238	248	228
Plasma homocysteine, µmol/L	M	9.73	9.72	9.92	9.77	10.3	10.5	10.8
	E	11.7	12.0	12.1	12.0	12.5	12.9	13.7
Plasma methionine, µmol/L	M	24.7	25.9	23.4	21.1	21.0	20.1	20.5
	E	24.7	25.4	23.8	21.1	20.6	19.8	20.0
Plasma serine, µmol/L	M	120	121	116	112	113	113	115
	E	115	116	113	108	106	108	102
**Lipid-solube vitamins**								
Plasma retinol, µmol/L	M	2.08	2.13	2.15	2.15	2.11	2.11	2.12
	E	2.10	2.17	2.16	2.19	2.20	2.16	2.26
Plasma 25-OH-vitD, nmol/L	M	61.3	62.5	62.6	64.4	64.7	62.2	60.6
	E	66.3	66.9	66.8	66.8	66.6	64.7	70.7
Plasma α-tocopherol, µmol/L	M	34.4	34.0	34.2	35.1	34.7	34.8	34.9
	E	38.4	37.8	38.3	39.2	38.5	39.7	38.6
Plasma phylloquinone, nmol/L	M	1.64	1.62	1.58	1.46	1.48	1.53	1.32

	E	1.78	1.74	1.62	1.54	1.55	1.47	1.24
**B-vitamin biomarkers**								
Plasma thiamine, nmol/L	M	3.12	3.21	3.00	2.94	2.65	2.30	2.39
	E	3.69	3.72	3.52	3.30	3.17	3.08	3.04
Plasma TMP, nmol/L	M	7.74	8.01	7.40	7.04	6.96	6.66	6.47
	E	7.44	7.49	7.29	6.91	6.90	6.56	6.22
Plasma riboflavin, nmol/L	M	14.2	13.5	12.9	13.2	12.6	12.4	14.2
	E	16.3	16.9	16.3	15.9	16.4	17.0	18.6
Plasma FMN, nmol/L	M	13.0	12.5	13.1	14.2	14.7	15.4	16.3
	E	12.5	11.7	11.9	12.9	14.0	14.5	14.2
Plasma nicotinamide, nmol/L	M	395	390	403	421	394	402	382
	E	328	380	374	382	361	415	421
Plasma methyl nicotinamide, nmol/L	M	82.4	87.4	83.2	86.0	79.9	75.8	64.0
	E	88.8	103	94.9	97.8	93.2	99.7	103
Plasma pyridoxal, nmol/L	M	13.1	13.3	12.5	12.1	11.9	11.8	11.0
	E	15.5	15.2	14.2	13.7	13.5	13.7	15.8
Plasma PLP, nmol/L	M	55.1	57.5	53.7	52.9	51.1	51.5	46.7
	E	55.1	55.0	53.7	51.2	51.3	50.8	55.9
Plasma 4-pyridoxic acid, nmol/L	M	23.1	23.5	22.9	23.7	22.9	21.1	19.2
	E	33.5	32.6	31.3	31.8	31.0	31.4	35.0
Plasma folate, nmol/L	M	7.23	7.02	6.84	7.01	6.78	6.64	6.62
	E	7.01	7.12	7.05	7.21	7.09	7.39	6.42
Plasma cobalamin, pmol/L	M	353	355	355	363	355	381	367
	E	358	350	348	349	350	361	322
Plasma MMA, µmol/L	M	0.18	0.18	0.18	0.17	0.17	0.16	0.17
	E	0.22	0.22	0.22	0.22	0.21	0.21	0.23

[1]All values are presented as geometric means. An overview of missing observations at each timepoint for each of the metabolites can be found in Supplementary Table 4. [2]Middle-aged group (aged 46-49 years) / Elderly group (aged 70-74 years). **Abbreviations**: FMN, flavin mononucleotide; HDL, High-density lipoprotein; LDL, Low-density lipoprotein; MMA, methyl-malonic acid; PLP, pyridoxal 5’-phosphate; TMP, thiamine monophosphate

Supplementary Table 3. The geometric means of metabolite concentrations the first seven hours after a meal in males (*n* = 2541) and females (*n* = 3293) in the Hordaland Health Study 1997-1999[1]

**Hours after meal**		**0-<1**	**1-<2**	**2-<3**	**3-<4**	**4-<5**	**5-<6**	**6-<7**
***n* participants**	M[2]	235	743	647	452	313	111	40
	F[2]	302	885	886	668	388	123	41
**Serum glucose, mmol/L**	M	5.98	5.65	5.39	5.17	5.05	5.01	4.94
	F	5.81	5.23	5.19	4.94	5.02	5.12	5.08
**Amino acids**								
Plasma alanine, µmol/L	M	410	435	409	377	361	347	328
	F	387	405	379	346	339	321	335
Plasma arginine, µmol/L	M	50.1	51.8	49.3	46.3	44.5	43.7	43.6
	F	46.1	49.2	47.3	43.7	43.3	39.7	41.6
Plasma asparagine, µmol/L	M	50.8	50.9	47.8	44.5	44.4	43.1	42.1
	F	50.2	50.7	47.7	44.5	43.9	43.1	42.8
Plasma aspartic acid, µmol/L	M	9.14	9.41	9.00	8.52	8.32	8.43	7.95
	F	8.35	8.52	8.25	7.78	7.37	7.24	7.67
Plasma glutamic acid, µmol/L	M	105	102	101	99.6	98.6	102	96.8
	F	83.6	84.7	85.2	82.5	83.4	80.5	87.2
Plasma glutamine, µmol/L	M	524	534	523	510	520	515	524
	F	513	518	512	503	512	508	497
Plasma histidine, µmol/L	M	84.8	85.1	82.4	78.6	79.0	77.7	75.6
	F	82.0	82.2	79.1	75.7	75.9	73.2	74.2
Plasma isoleucine, µmol/L	M	83.5	85.5	79.1	71.5	71.8	72.6	65.6
	F	71.4	70.8	67.5	62.0	62.9	59.3	59.0
Plasma leucine, µmol/L	M	151	154	143	131	131	132	122
	F	128	128	122	113	114	109	111
Plasma lysine, µmol/L	M	187	197	188	176	174	165	157
	F	177	184	178	167	165	156	155
Plasma phenylalanine, µmol/L	M	66.4	68.3	65.0	59.6	59.7	57.5	58.2
	F	63.3	64.4	61.6	57.8	57.1	54.9	57.5
Plasma proline, µmol/L	M	232	241	234	215	207	207	197
	F	203	209	200	183	177	160	169
Plasma threonine, µmol/L	M	129	134	128	121	119	119	121
	F	129	131	125	117	117	112	113
Plasma tryptophan, µmol/L	M	73.9	74.6	71.4	66.5	64.5	64.8	61.8
	F	66.3	69.1	65.6	60.4	58.9	55.8	59.3
Plasma tyrosine, µmol/L	M	68.5	71.4	67.7	64.0	61.9	60.4	58.1

	F	65.0	68.4	66.3	63.1	61.1	59.0	57.8
Plasma valine, µmol/L	M	279	285	275	260	261	257	240
	F	246	247	243	230	232	222	224
**Blood lipis**								
Serum total cholesterol, mmol/L	M	5.60	5.73	5.76	5.79	5.82	5.82	5.89
	F	5.78	5.88	6.03	6.10	6.15	5.97	6.04
Serum LDL-cholesterol, mmol/L	M	3.60	3.73	3.76	3.76	3.85	3.87	3.89
	F	3.65	3.70	3.85	3.93	3.93	3.79	4.00
Serum HDL-cholesterol, mmol/L	M	1.13	1.14	1.14	1.18	1.16	1.11	1.22
	F	1.37	1.42	1.41	1.43	1.43	1.44	1.29
Serum triglycerides, mmol/L	M	1.76	1.79	1.75	1.69	1.60	1.66	1.49
	F	1.47	1.42	1.42	1.43	1.44	1.34	1.37
**One-carbon metabolites**								
Plasma betaine, µmol/L	M	44.1	44.8	44.4	42.2	42.0	40.3	43.6
	F	33.2	34.6	35.3	33.3	33.1	30.3	30.2
Plasma choline, µmol/L	M	10.2	10.6	10.3	10.2	9.92	9.32	9.39
	F	9.40	0.52	9.31	0.13	8.80	8.29	8.35
Plasma cysteine, µmol/L	M	302	302	307	308	315	310	318
	F	292	289	298	300	309	317	313
Plasma cystathionine, µmol/L	M	0.23	0.25	0.26	0.24	0.25	0.21	0.19
	F	0.19	0.20	0.22	0.20	0.21	0.17	0.18
Plasma dimethylglycine, µmol/L	M	4.71	4.88	4.77	4.65	4.64	4.65	4.61
	F	4.24	4.33	4.28	4.24	4.16	3.88	4.17
Plasma glycine, µmol/L	M	233	234	232	226	222	219	219
	F	269	276	271	265	254	266	254
Plasma homocysteine, µmol/L	M	11.3	11.6	11.9	11.8	13.2	12.8	12.7
	F	9.57	9.91	10.4	10.4	11.0	10.7	11.7
Plasma methionine, µmol/L	M	26.5	27.6	25.3	22.5	21.8	21.0	20.5
	F	23.3	24.2	22.4	20.2	20.0	18.9	20.0
Plasma serine, µmol/L	M	114	114	111	105	105	108	107
	F	122	122	118	112	112	112	109
**Lipid-solube vitamins**								
Plasma retinol, µmol/L	M	2.21	2.30	2.30	2.32	2.30	2.25	2.25
	F	2.00	2.03	2.06	2.07	2.07	2.04	2.13
Plasma 25-OH-vitD, nmol/L	M	60.8	64.1	64.4	65.4	68.1	62.3	64.3
	F	64.4	64.7	65.1	65.8	64.1	64.6	66.5
Plasma α-tocopherol, µmol/L	M	34.5	34.3	34.8	35.4	35.3	35.7	35.4
	F	36.6	36.8	37.4	38.6	38.3	38.7	38.0
Plasma phylloquinone, nmol/L	M	1.75	1.82	1.72	1.61	1.62	1.61	1.31

	F	1.64	1.56	1.52	1.43	1.45	1.41	1.26
**B-vitamin biomarkers**								
Plasma thiamine, nmol/L	M	2.76	2.97	2.86	2.69	2.53	2.30	2.29
	F	3.77	3.85	3.60	3.46	3.34	3.06	3.15
Plasma TMP, nmol/L	M	6.80	7.01	6.55	6.21	6.24	6.06	5.85
	F	8.36	8.47	7.97	7.50	7.53	7.12	6.87
Plasma riboflavin, nmol/L	M	13.0	13.8	14.0	12.5	13.0	11.9	13.9
	F	16.3	15.8	15.0	16.2	16.4	17.5	18.8
Plasma FMN, nmol/L	M	12.8	12.0	12.3	12.7	13.5	14.0	14.4
	F	12.9	12.2	12.6	14.0	15.0	15.8	16.1
Plasma nicotinamide, nmol/L	M	366	377	383	392	367	423	395
	F	377	392	391	404	379	396	408
Plasma methyl nicotinamide, nmol/L	M	77.6	85.2	81.8	82.5	80.9	80.3	79.1
	F	90.0	102	94.8	99.2	93.5	94.4	96.1
Plasma pyridoxal, nmol/L	M	13.5	13.8	13.1	12.4	12.2	12.0	12.2
	F	14.1	14.3	13.5	13.3	13.3	13.4	14.2
Plasma PLP, nmol/L	M	54.0	54.8	52.7	51.6	50.3	50.1	49.3
	F	56.0	57.7	54.4	52.2	51.9	52.1	52.8
Plasma 4-pyridoxic acid, nmol/L	M	24.8	25.6	26.2	26.9	26.3	25.4	23.4
	F	27.0	28.5	27.5	28.4	28.5	26.5	28.4
Plasma folate, nmol/L	M	6.34	6.61	6.52	6.40	6.40	5.88	6.43
	F	7.87	7.48	7.29	7.64	7.46	8.24	6.61
Plasma cobalamin, pmol/L	M	354	343	347	351	343	349	345
	F	354	362	355	359	359	391	343
Plasma MMA, µmol/L	M	0.18	0.20	0.20	0.20	0.19	0.20	0.20
	F	0.19	0.20	0.20	0.19	0.19	0.17	0.19

[1]All values are presented as geometric means. An overview of missing observations at each timepoint for each of the metabolites can be found in Supplementary Table 4. [2]Males/females **Abbreviations**: FMN, flavin mononucleotide; HDL, High-density lipoprotein; LDL, Low-density lipoprotein; MMA, methyl-malonic acid; PLP, pyridoxal 5'-phosphate; TMP, thiamine monophosphate

Supplementary Table 4. The number of missing values for each metabolite at each timepoint in the Hordaland Health Study 1997-1999

**Hours after meal, h**	**1**	**2**	**3**	**4**	**5**	**6**	**7**
n	537	1628	1533	1120	701	234	81
Serum glucose, mmol/L	0	0	0	0	0	0	0
**Amino acids**							
Plasma alanine, µmol/L	13	23	27	20	13	1	2
Plasma arginine, µmol/L	1	1	2	2	0	0	0
Plasma asparagine, µmol/L	13	27	29	27	18	2	2
Plasma aspartic acid, µmol/L	13	23	27	19	23	1	2
Plasma glutamic acid, µmol/L	13	23	27	19	23	1	2
Plasma glutamine, µmol/L	13	23	28	25	15	2	2
Plasma histidine, µmol/L	13	23	27	20	13	1	2
Plasma isoleucine, µmol/L	13	23	27	20	13	1	2
Plasma leucine, µmol/L	13	23	27	20	13	1	2
Plasma lysine, µmol/L	13	23	27	20	13	1	2
Plasma phenylalanine, µmol/L	14	23	28	25	15	2	2
Plasma proline, µmol/L	14	28	30	24	15	1	2
Plasma threonine, µmol/L	14	24	28	25	15	2	2
Plasma tryptophan, µmol/L	6	9	16	11	4	2	0
Plasma tyrosine, µmol/L	13	23	28	25	15	2	2
Plasma valine, µmol/L	13	23	27	20	13	1	2
**Blood lipids**							
Serum total cholesterol, mmol/L	0	0	0	0	0	0	0
Serum LDL-cholesterol, mmol/L	0	0	0	0	0	0	0
Serum HDL-cholesterol, mmol/L	0	0	0	0	0	0	0
Serum triglycerides, mmol/L	0	0	0	0	0	0	0
**One-carbon metabolites**							
Plasma betaine, µmol/L	1	1	2	2	0	0	0
Plasma choline, µmol/L	1	1	2	2	0	0	0
Plasma cysteine, µmol/L	5	17	17	13	8	3	1
Plasma cystathionine, µmol/L	1	1	2	2	0	1	0
Plasma dimethylglycine, µmol/L	1	1	2	2	0	0	0
Plasma glycine, µmol/L	13	23	27	20	13	1	2
Plasma homocysteine, µmol/L	1	1	2	2	0	0	0
Plasma methionine, µmol/L	1	1	2	2	0	0	0
Plasma serine, µmol/L	13	23	27	20	13	1	2
**Lipid-soluble vitamins**							
Plasma retinol, µmol/L	13	23	30	20	13	1	2
Plasma 25-OH-vitD, nmol/L	13	31	31	21	14	2	2
Plasma α-tocopherol, µmol/L	13	23	30	20	13	1	2
Plasma phylloquinone, nmol/L	48	144	147	121	95	31	16
**B-vitamin biomarkers**							
Plasma thiamine, nmol/L	13	45	59	49	25	7	2
Plasma TMP, nmol/L	13	45	60	49	25	7	2
Plasma riboflavin, nmol/L	6	9	16	11	6	3	1

Plasma FMN, nmol/L	6	9	16	11	6	3	1
Plasma nicotinamide, nmol/L	13	46	59	50	26	8	2
Plasma methyl nicotinamide, nmol/L	13	45	60	49	25	8	2
Plasma pyridoxal, nmol/L	6	9	16	11	4	2	0
Plasma PLP, nmol/L	6	9	16	11	4	2	0
Plasma 4-pyridoxic acid, nmol/L	6	9	16	11	4	2	0
Plasma folate, nmol/L	1	8	4	6	4	0	0
Plasma cobalamin, pmol/L	0	3	2	3	0	0	0
Plasma MMA, µmol/L	28	54	56	40	40	9	7

Abbreviations: HDL, High density lipoprotein; LDL, Low-density lipoprotein

Supplementary Table 5. The number of missing observations at each timepoint in middle-aged and elderly adults, and among males and females in the Hordaland Health Study 1997-1999

	**Age cohorts**														**Sex**													
	**Middle-aged group,** aged 46-49 years, *n* = 2960							**Elderly group,** aged 70-74 years, *n* = 2874							**Males,** *n* = 2541							**Females,** *n* = 3293						
**Hours after meal**	**1**	**2**	**3**	**4**	**5**	**6**	**7**	**1**	**2**	**3**	**4**	**5**	**6**	**7**	**1**	**2**	**3**	**4**	**5**	**6**	**7**	**1**	**2**	**3**	**4**	**5**	**6**	**7**
***n* participants**	369	916	731	514	276	113	41	168	712	803	606	425	121	40	235	743	647	452	313	111	40	302	885	886	668	388	123	41
Glucose	0	0	0	0	0	0	0	0	0	0	0	0	0	0	0	0	0	0	0	0	0	0	0	0	0	0	0	0
**Amino acids**																												
Alanine	10	16	12	10	5	1	1	3	7	15	10	8	0	1	8	17	15	16	9	1	1	5	6	12	4	4	0	1
Arginine	1	1	0	1	0	0	0	0	0	2	1	0	0	0	1	1	2	2	0	0	0	0	0	0	0	0	0	0
Asparagine	10	17	13	15	7	2	1	3	10	16	12	11	0	1	8	18	16	19	10	1	1	5	9	13	8	8	1	1
Aspartic acid	10	16	12	9	15	1	1	3	7	15	10	8	0	1	8	17	15	15	9	1	1	5	6	12	4	4	0	1
Glutamic acid	10	16	12	9	15	1	1	3	7	15	10	8	0	1	8	17	15	16	9	1	1	5	6	12	4	4	0	1
Glutamine	10	16	13	15	7	2	1	3	7	15	10	8	0	1	8	17	15	16	9	1	1	5	6	12	4	4	0	1
Histidine	10	16	12	10	5	1	1	3	7	15	10	8	0	1	8	17	15	16	9	1	1	5	6	12	4	4	0	1
Isoleucine	10	16	12	10	5	1	1	3	7	15	10	8	0	1	8	17	15	16	9	1	1	5	6	12	4	4	0	1
Leucine	10	16	12	10	5	1	1	3	7	15	10	8	0	1	8	17	15	16	9	1	1	5	6	12	4	4	0	1
Lysine	10	16	12	10	5	1	1	3	7	15	10	8	0	1	8	17	15	16	9	1	1	5	6	12	4	4	0	1
Phenylalanine	11	16	13	15	7	2	1	3	7	15	10	8	0	1	8	17	15	16	9	1	1	6	6	12	4	4	0	1
Proline	11	20	13	11	6	1	1	3	8	17	13	9	0	1	8	17	15	20	10	1	1	6	8	15	7	5	0	1
Threonine	10	16	13	15	7	2	1	3	7	15	10	8	0	1	8	17	15	16	9	1	1	5	6	12	4	4	0	1
Tryptophan	6	8	9	6	1	1	0	0	1	7	5	3	1	0	4	7	8	9	4	1	0	2	2	8	2	0	1	0
Tyrosine	10	16	13	15	7	2	1	3	7	15	10	8	0	1	8	17	15	16	9	1	1	5	6	12	4	4	0	1
Valine	10	16	12	10	5	1	1	3	7	15	10	8	0	1	8	17	15	16	9	1	1	5	6	12	4	4	0	1
**Lipids**																												
Cholesterol	0	0	0	0	0	0	0	0	0	0	0	0	0	0	0	0	0	0	0	0	0	0	0	0	0	0	0	0

LDL-C	0	0	0	0	0	0	0	0	0	0	0	0	0	0	0	0	0	0	0	0	0	0	0	0	0	0	0	0
HDL-C	0	0	0	0	0	0	0	0	0	0	0	0	0	0	0	0	0	0	0	0	0	0	0	0	0	0	0	0
Triglycerides	0	0	0	0	0	0	0	0	0	0	0	0	0	0	0	0	0	0	0	0	0	0	0	0	0	0	0	0
**One-carbon metabolites**																												
Betaine	1	1	0	1	0	0	0	0	0	2	1	0	0	0	1	1	2	2	0	0	0	0	0	0	0	0	0	0
Choline	1	1	0	1	0	0	0	0	0	2	1	0	0	0	1	1	2	2	0	0	0	0	0	0	0	0	0	0
Cysteine	1	1	0	1	0	0	0	4	16	17	12	8	3	1	3	8	8	6	3	1	1	2	9	9	7	5	2	0
Cystathionine	1	1	1	1	0	0	0	0	0	2	1	0	1	0	3	8	8	6	3	1	1	0	0	1	0	0	1	0
DMG	1	1	0	1	0	0	0	0	0	2	1	0	0	0	1	1	2	2	0	0	0	0	0	0	0	0	0	0
Glycine	10	16	12	10	5	1	1	3	7	15	10	8	0	1	8	17	15	16	9	1	1	5	6	12	4	4	0	1
Homocysteine	1	1	0	1	0	0	0	0	0	2	1	0	0	0	1	1	2	2	0	0	0	0	0	0	0	0	0	0
Methionine	1	1	0	1	0	0	0	0	0	2	1	0	0	0	1	1	2	2	0	0	0	0	0	0	0	0	0	0
Serine	10	16	12	10	5	1	1	3	7	15	10	8	0	1	8	17	15	16	9	1	1	5	6	12	4	4	0	1
**Lipid-soluble vitamins**																												
Retinol	9	17	14	10	5	1	1	4	7	16	10	8	0	1	4	6	14	4	4	0	1	9	18	16	16	9	1	1
25-OH-vitD	9	23	15	11	5	2	1	4	8	16	10	9	0	1	4	10	14	4	5	0	1	9	21	17	17	9	2	1
α-tocopherol	9	17	14	10	5	1	1	4	7	16	10	8	0	1	4	6	14	4	4	0	1	9	18	16	16	9	1	1
Phylloquinone	40	94	81	69	43	19	8	8	50	66	52	52	12	8	25	88	99	74	58	16	12	23	56	48	47	37	15	4
**B-vitamin biomarkers**																												
Thiamine	12	24	28	24	10	5	1	1	21	31	25	15	2	1	6	27	30	28	13	5	1	7	18	29	21	12	2	1
TMP	12	24	29	24	10	5	1	1	21	31	25	15	2	1	6	27	30	28	13	5	1	7	18	30	21	12	2	1
Riboflavin	6	8	9	6	1	1	0	0	1	7	5	5	2	1	4	7	8	9	4	1	0	2	2	8	2	0	1	0
FMN	6	8	9	6	1	1	0	0	1	7	5	5	2	1	4	7	8	9	4	1	0	2	2	8	2	0	1	0
Nicotinamide	12	24	28	25	10	6	1	1	22	31	25	16	2	1	6	28	30	28	14	5	1	7	18	29	22	12	3	1
Methyl nicotinamide	12	24	29	24	10	6	1	1	21	31	25	15	2	1	6	27	30	28	13	5	1	7	18	29	21	12	2	1
Pyridoxal	6	8	9	6	1	1	0	0	1	7	5	3	1	0	4	7	8	9	4	1	0	2	2	8	2	0	1	0
PLP	6	8	9	6	1	1	0	0	1	7	5	3	1	0	4	7	8	9	4	1	0	2	2	8	2	0	1	0
4-pyridoxic acid	6	8	9	6	1	1	0	0	1	7	5	3	1	0	4	7	8	9	4	1	0	2	2	8	2	0	1	0

Folate	1	4	2	1	0	0	0	0	4	2	5	4	0	0	0	6	0	5	3	0	0	1	2	4	1	1	0	0
Cobalamin	0	2	1	1	0	0	0	0	1	1	2	0	0	0	0	3	0	3	0	0	0	0	0	2	0	0	0	0
MMA	22	38	34	20	21	4	5	6	16	22	20	19	5	2	17	26	28	27	21	7	3	11	28	28	13	19	2	4

Abbreviations: 25-OH-vitD, 25-hydroxyvitamin D; DMG, dimethylglycine; FMN, Flavin mononucleotide; HDL-C, High density lipoprotein cholesterol; HUSK, Hordaland Health Study; LDL-C, Low-density lipoprotein cholesterol; MMA, methylmalonic acid; PLP, Pyridoxal-5'-phosphate, TMP, Thiamine monophosphate

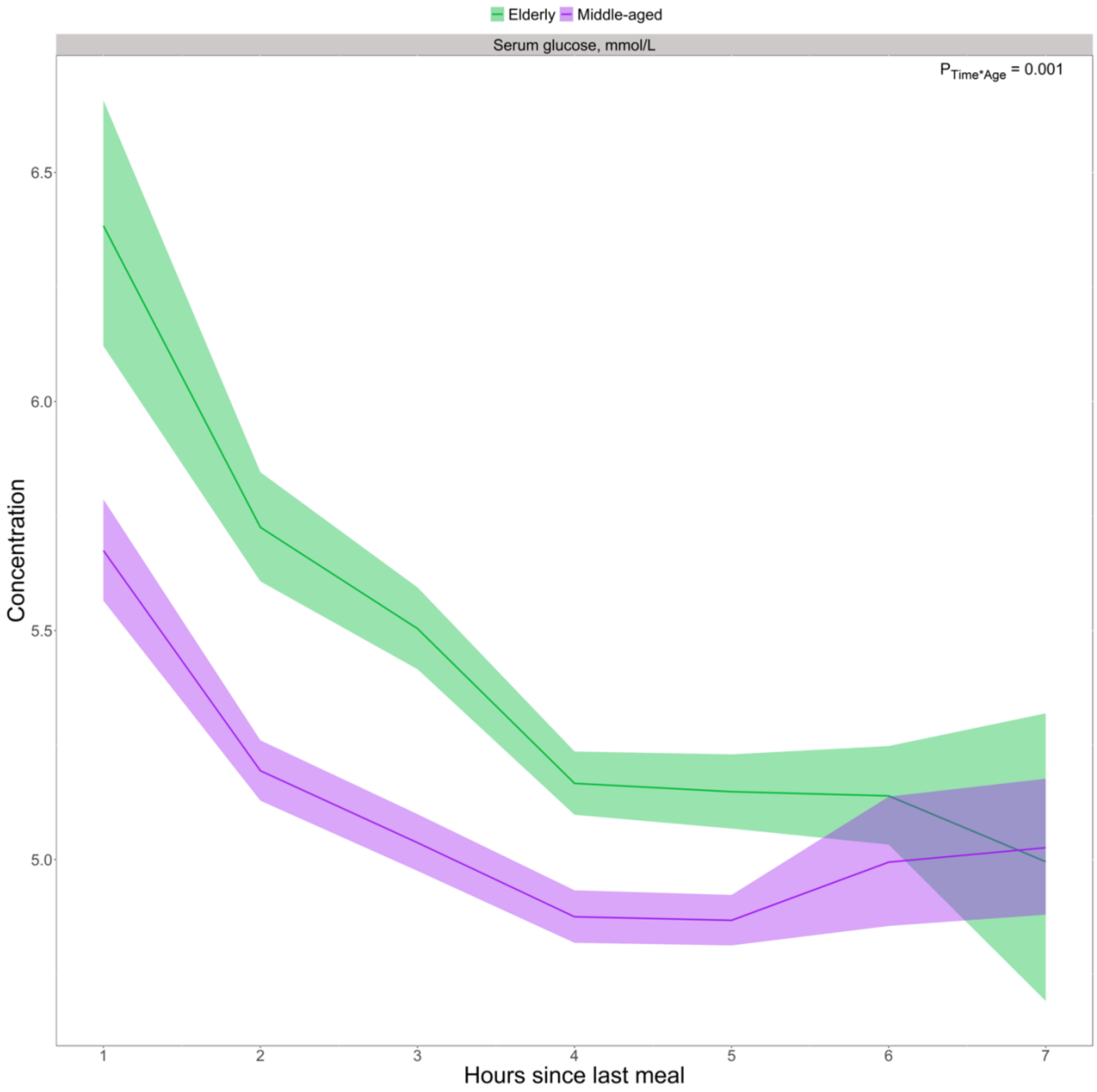

Supplementary Figure 1. The concentration of glucose as a function of time since the last meal in the middle-aged (*n* = 2960) and the elderly group (*n* = 2874) in the Hordaland Health Study 1997-1999. The solid line represents the geometric mean, while the colored shaded area represents the 95% geometric confidence intervals. Note that the origin of the y-axis ≠ 0. An overview of the number of observations at each timepoint is provided in Supplementary Table 4 and 5.

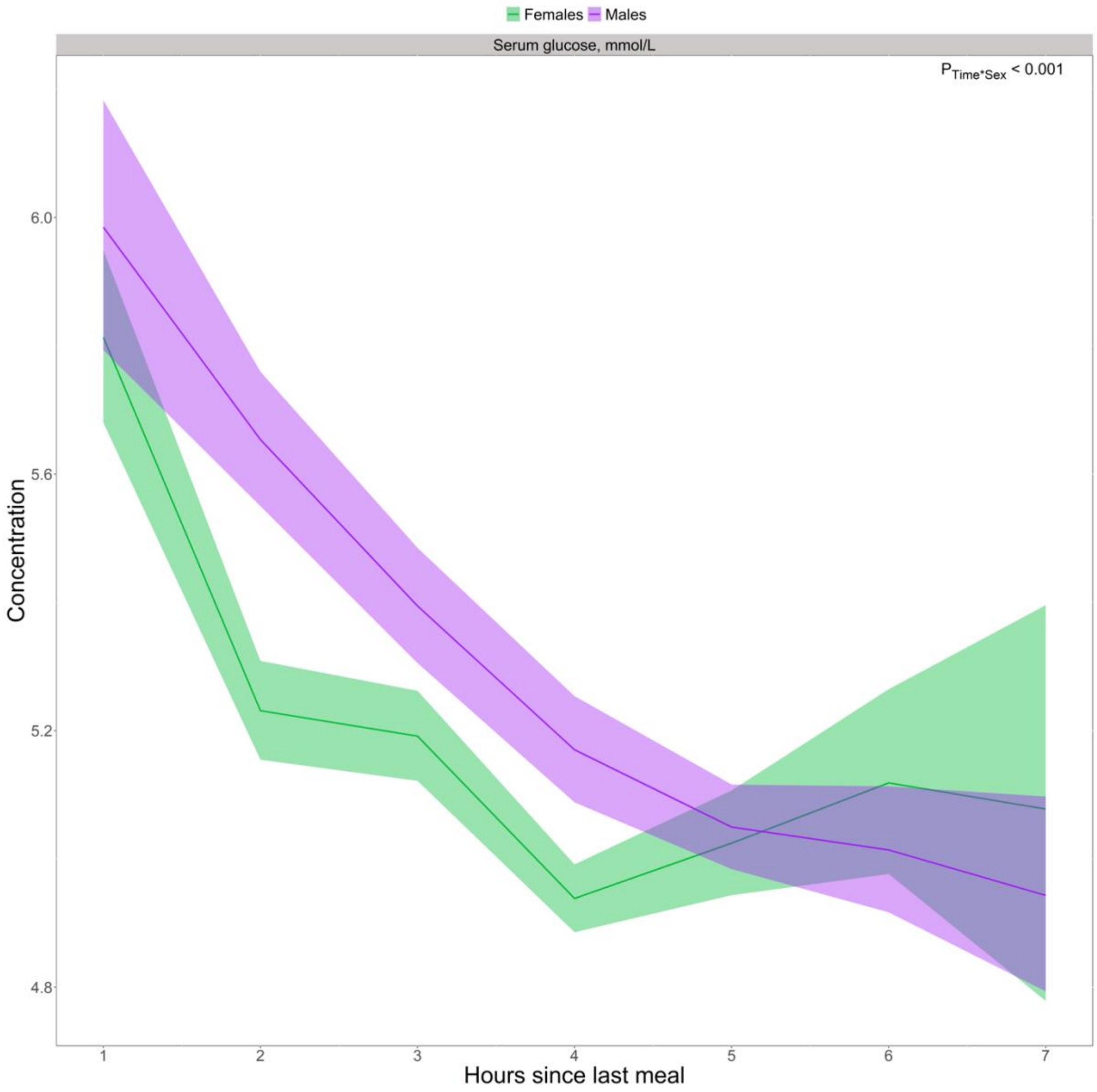

Supplementary Figure 2. The concentration of glucose as a function of time since the last meal in males (*n* = 2541) and females (*n* = 3293) in the Hordaland Health Study 1997-1999. The solid line represents the geometric mean, while the colored shaded area represents the 95% geometric confidence intervals. Note that the origin of the y-axis ≠ 0. An overview of the number of observations at each timepoint is provided in Supplementary Table 4 and 5.

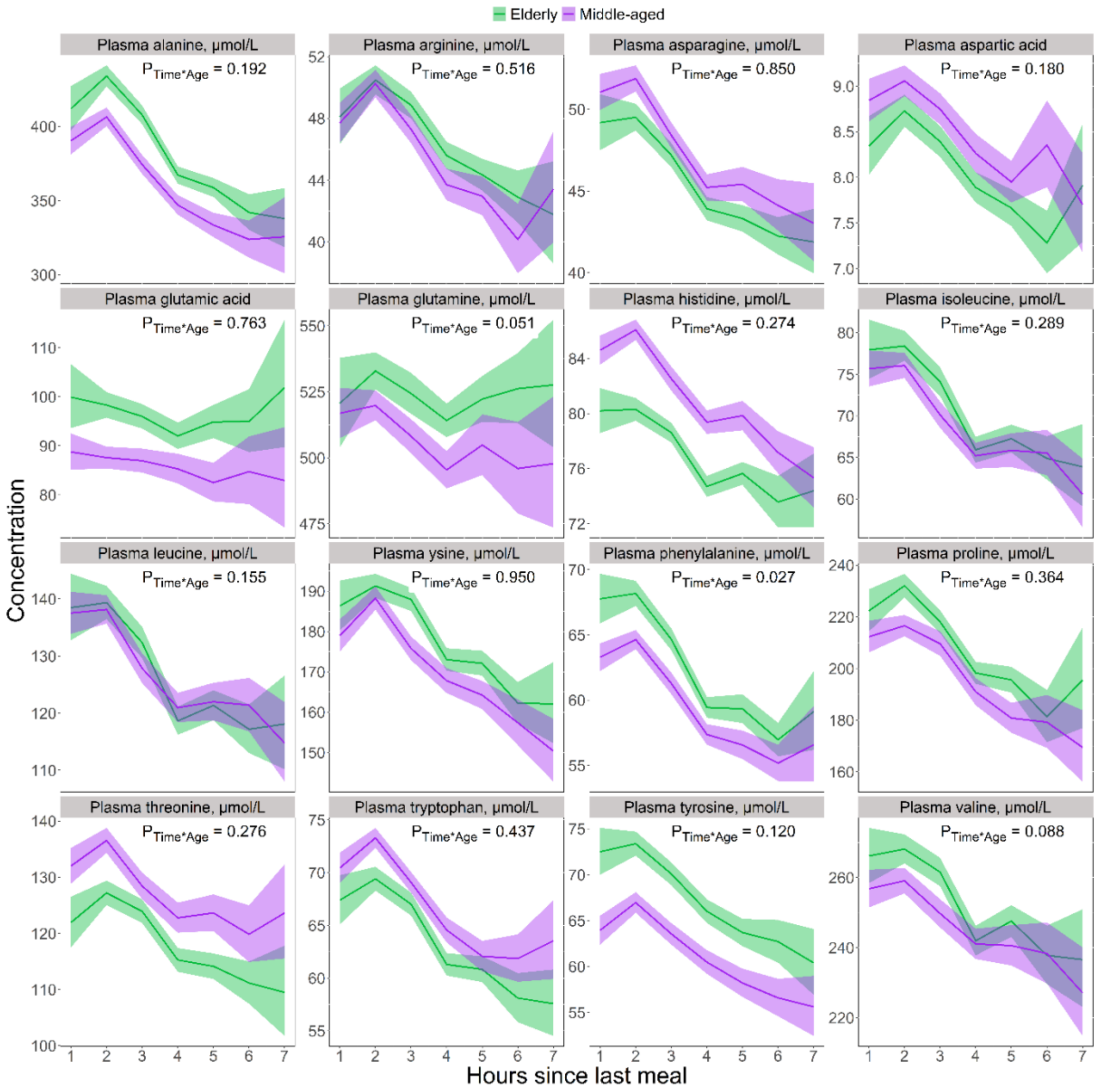

Supplementary Figure 3. The concentration of amino acids as a function of time since the last meal in the middle-aged (n = 2960) and the elderly group (n = 2874) in the Hordaland Health Study 1997-1999. The solid line represents the geometric mean, while the colored shaded area represents the 95% geometric confidence intervals. Note that the origin of the y-axis ≠ 0, and the y-axes are scales to be compatible with the metabolite concentration ranges. An overview of the number of observations at each timepoint is provided in Supplementary Table 4 and 5.

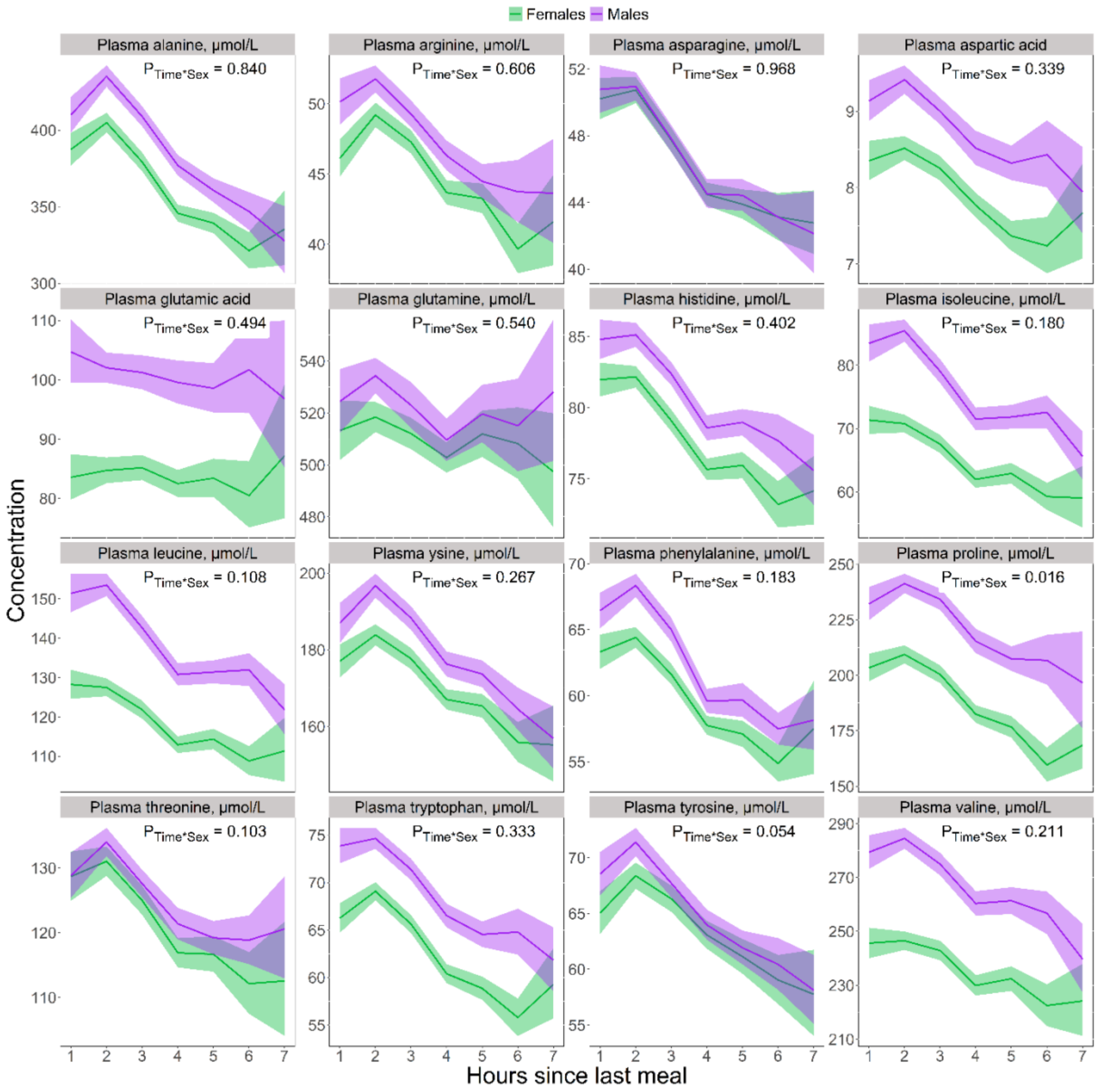

Supplementary Figure 4. The concentration of amino acids as a function of time since the last meal in males (*n* = 2541) and females (*n* = 3293) in the Hordaland Health Study 1997-1999. The solid line represents the geometric mean, while the colored shaded area represent the 95% geometric confidence intervals. Note that the origin of the y-axis ≠ 0, and the y-axes are scales to be compatible with the metabolite concentration ranges. An overview of the number of observations at each timepoint is provided in Supplementary Table 4 and 5

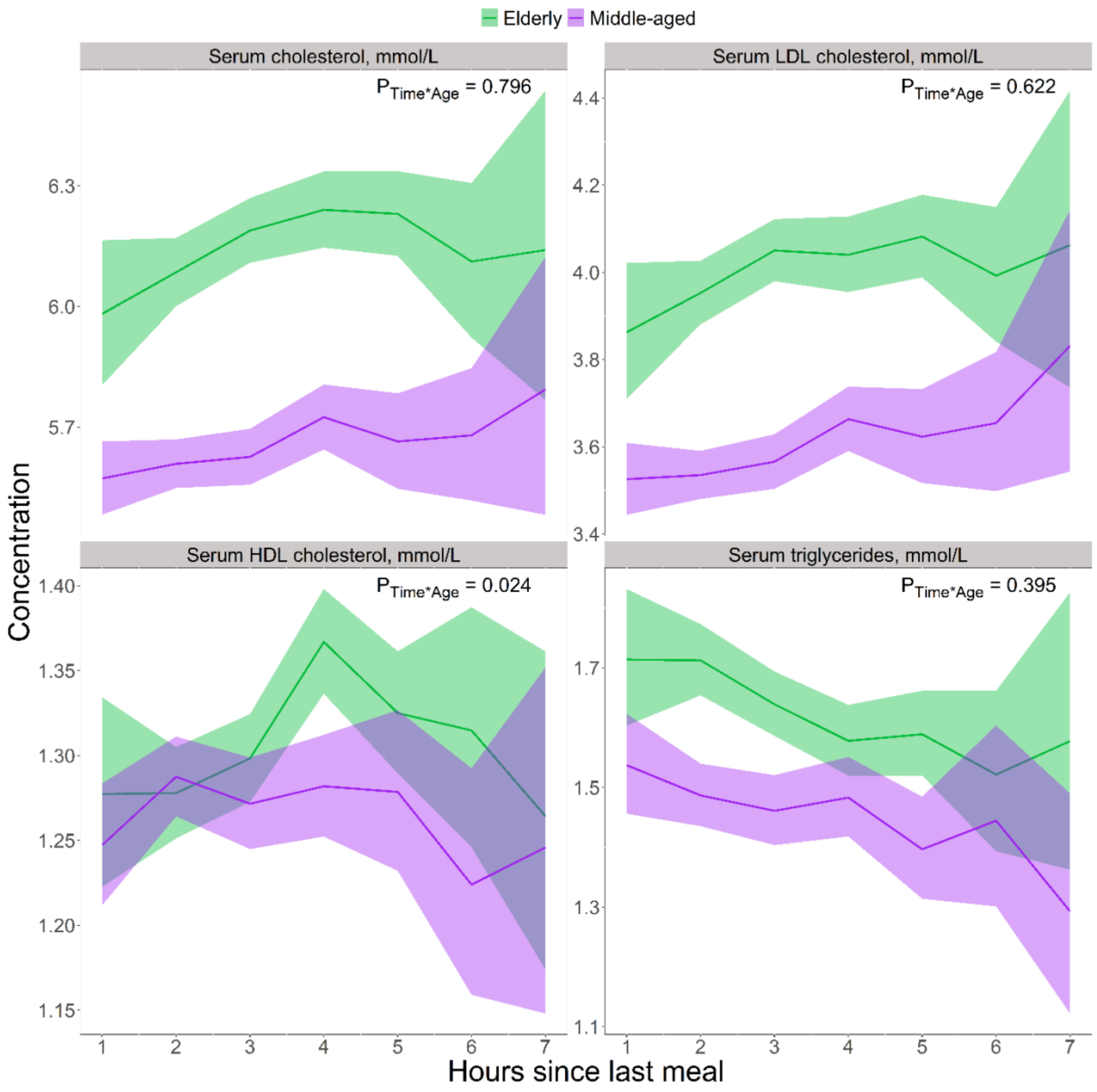

Supplementary Figure 5. The concentration of blood lipids as a function of time since the last meal in the middle-aged (*n* = 2960) and the elderly group (*n* = 2874) in the Hordaland Health Study 1997-1999. The solid line represents the geometric mean, while the colored shaded area represent the 95% geometric confidence intervals. Note that the origin of the y-axis ≠ 0, and the y-axes are scales to be compatible with the metabolite concentration ranges. An overview of the number of observations at each timepoint is provided in Supplementary Table 4 and 5. **Abbreviations**: HDL, High density lipoprotein; LDL, low density lipoprotein.

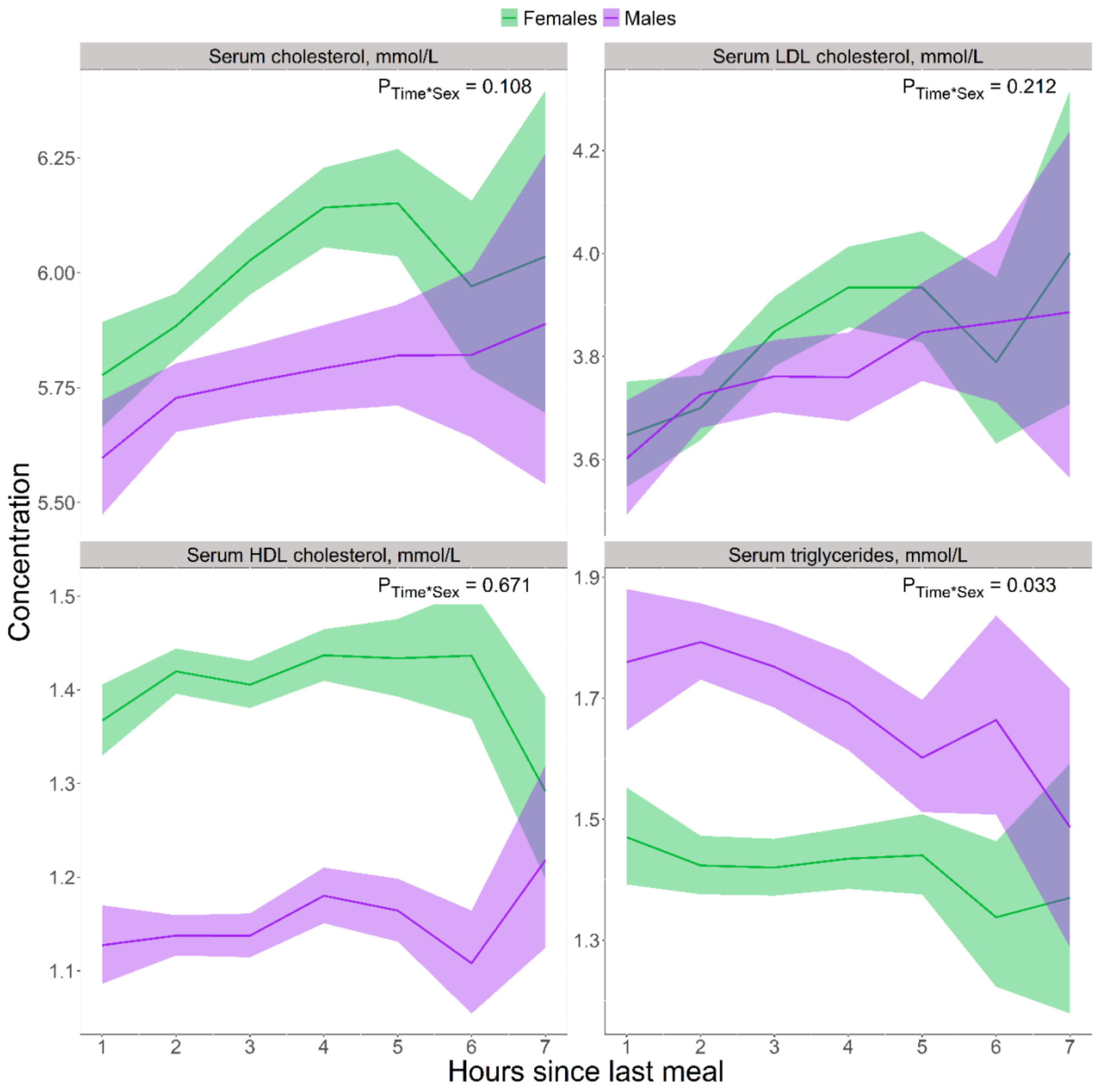

Supplementary Figure 6. The concentration of blood lipids as a function of time since the last meal in males (*n* = 2541) and females (*n* = 3293) in the Hordaland Health Study 1997-1999. The solid line represents the geometric mean, while the colored shaded area represent the 95% geometric confidence intervals. Note that the origin of the y-axis ≠ 0, and the y-axes are scales to be compatible with the metabolite concentration ranges. An overview of the number of observations at each timepoint is provided in Supplementary Table 4 and 5. **Abbreviations**: HDL, High density lipoprotein; LDL, low density lipoprotein

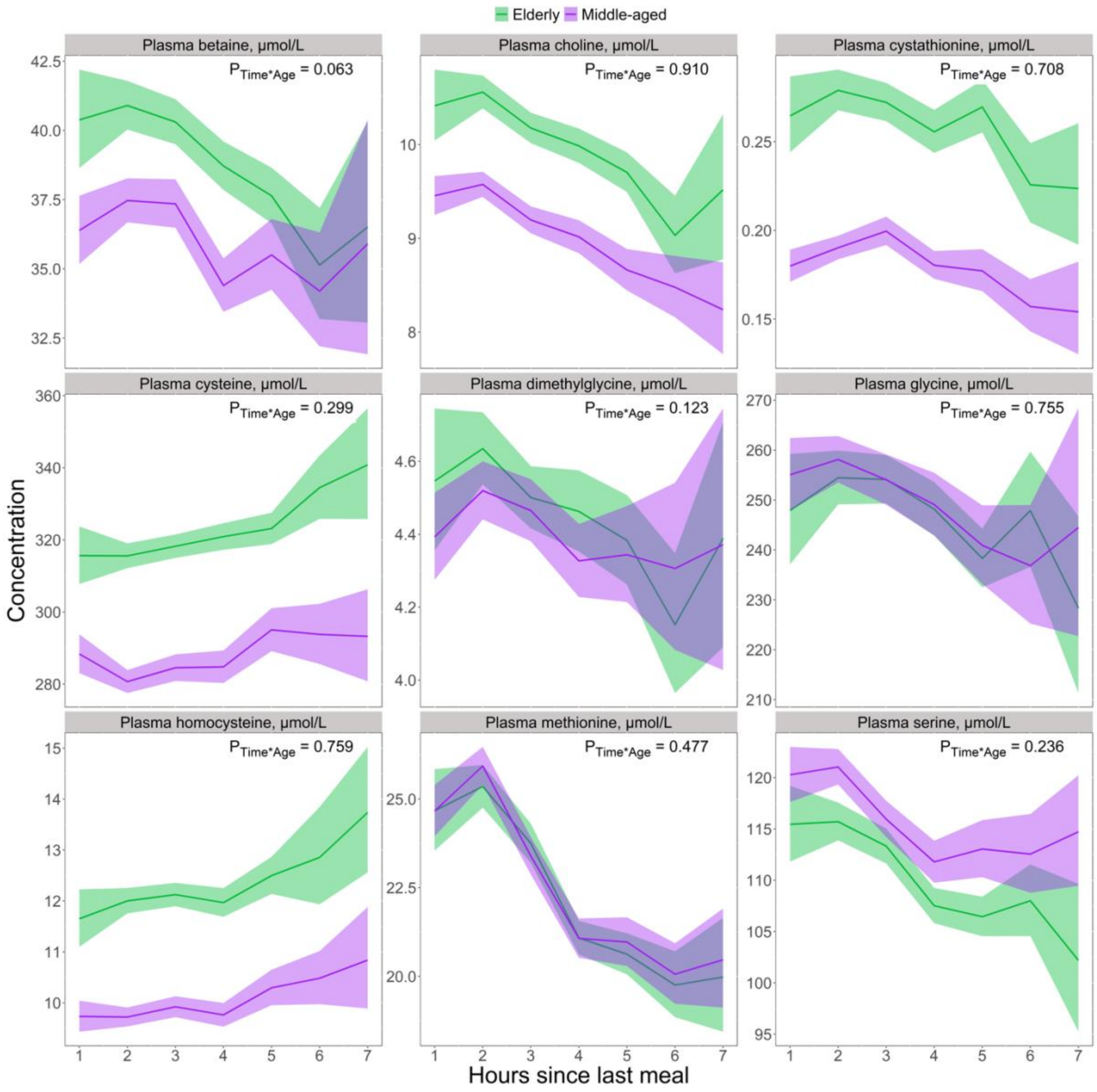

Supplementary Figure 7. The concentration of one-carbon metabolites as a function of time since the last meal in the middle-aged (*n* = 2960) and the elderly group (*n* = 2874) in the Hordaland Health Study 1997-1999. The solid line represents the geometric mean, while the colored shaded area represent the 95% geometric confidence intervals. Note that the origin of the y-axis ≠ 0, and the y-axes are scales to be compatible with the metabolite concentration ranges. An overview of the number of observations at each timepoint is provided in Supplementary Table 4 and 5.

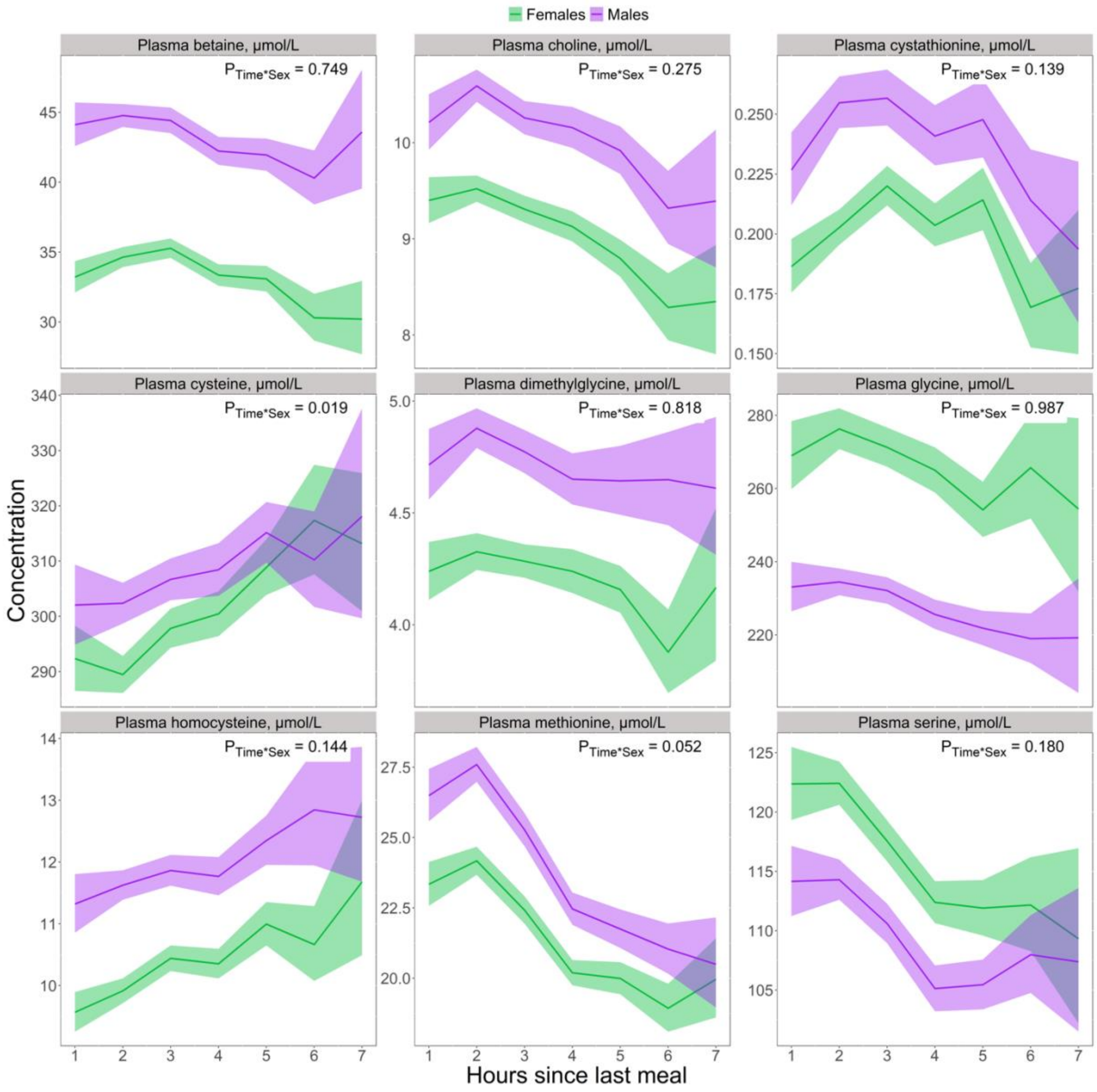

Supplementary Figure 8. The concentration of one-carbon metabolites as a function of time since the last meal in males (*n* = 2541) and females (*n* = 3293) in the Hordaland Health Study 1997-1999. The solid line represents the geometric mean, while the colored shaded area represent the 95% geometric confidence intervals. Note that the origin of the y-axis ≠ 0, and the y-axes are scales to be compatible with the metabolite concentration ranges. An overview of the number of observations at each timepoint is provided in Supplementary Table 4 and 5.

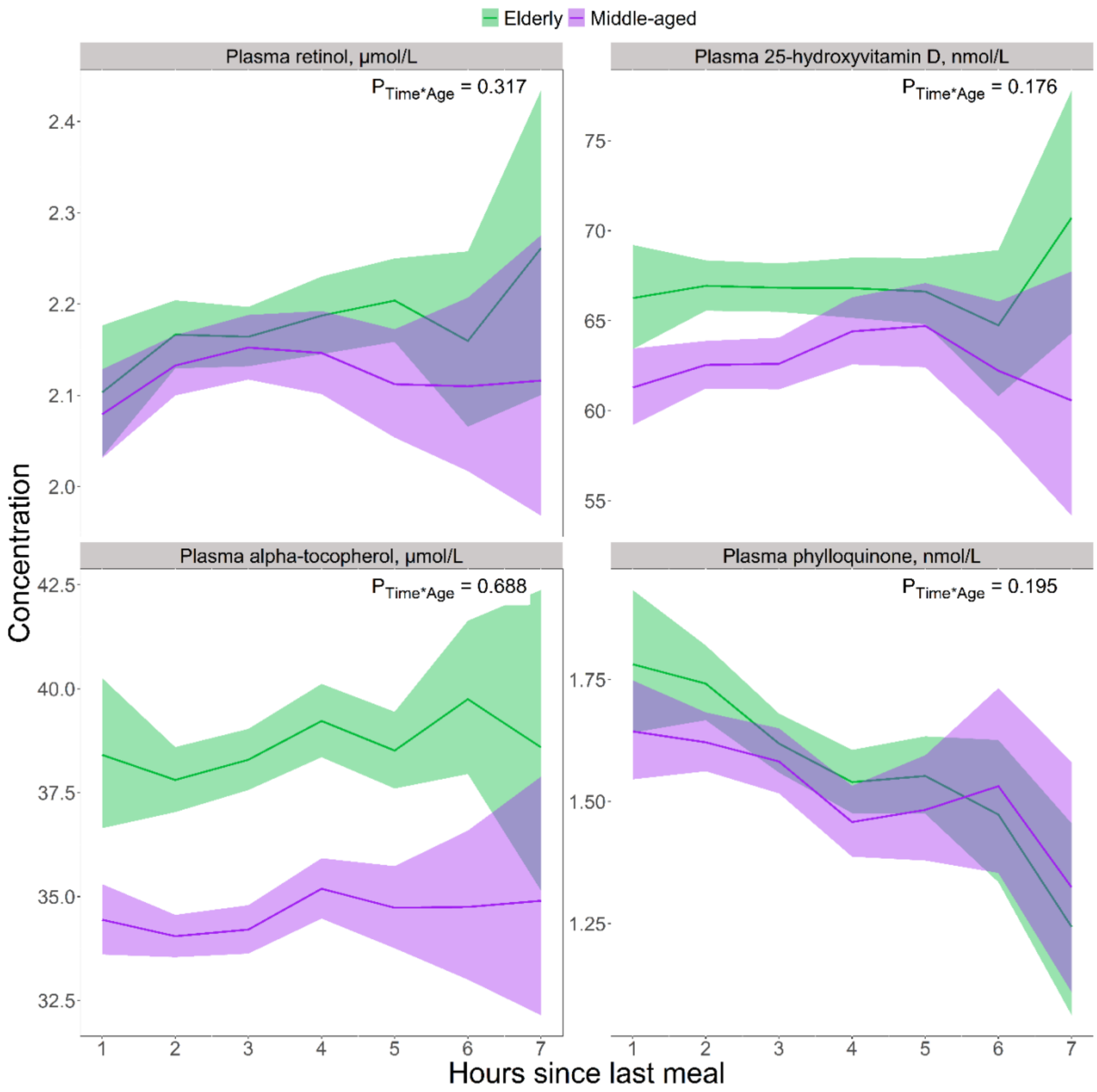

Supplementary Figure 9. The concentration of lipid-soluble vitamins as a function of time since the last meal in the middle-aged (*n* = 2960) and the elderly group (*n* = 2874) in the Hordaland Health Study 1997-1999. The solid line represents the geometric mean, while the colored shaded area represents the 95% geometric confidence intervals. Note that the origin of the y-axis ≠ 0, and the y-axes are scales to be compatible with the metabolite concentration ranges. An overview of the number of observations at each timepoint is provided in Supplementary Table 4 and 5.

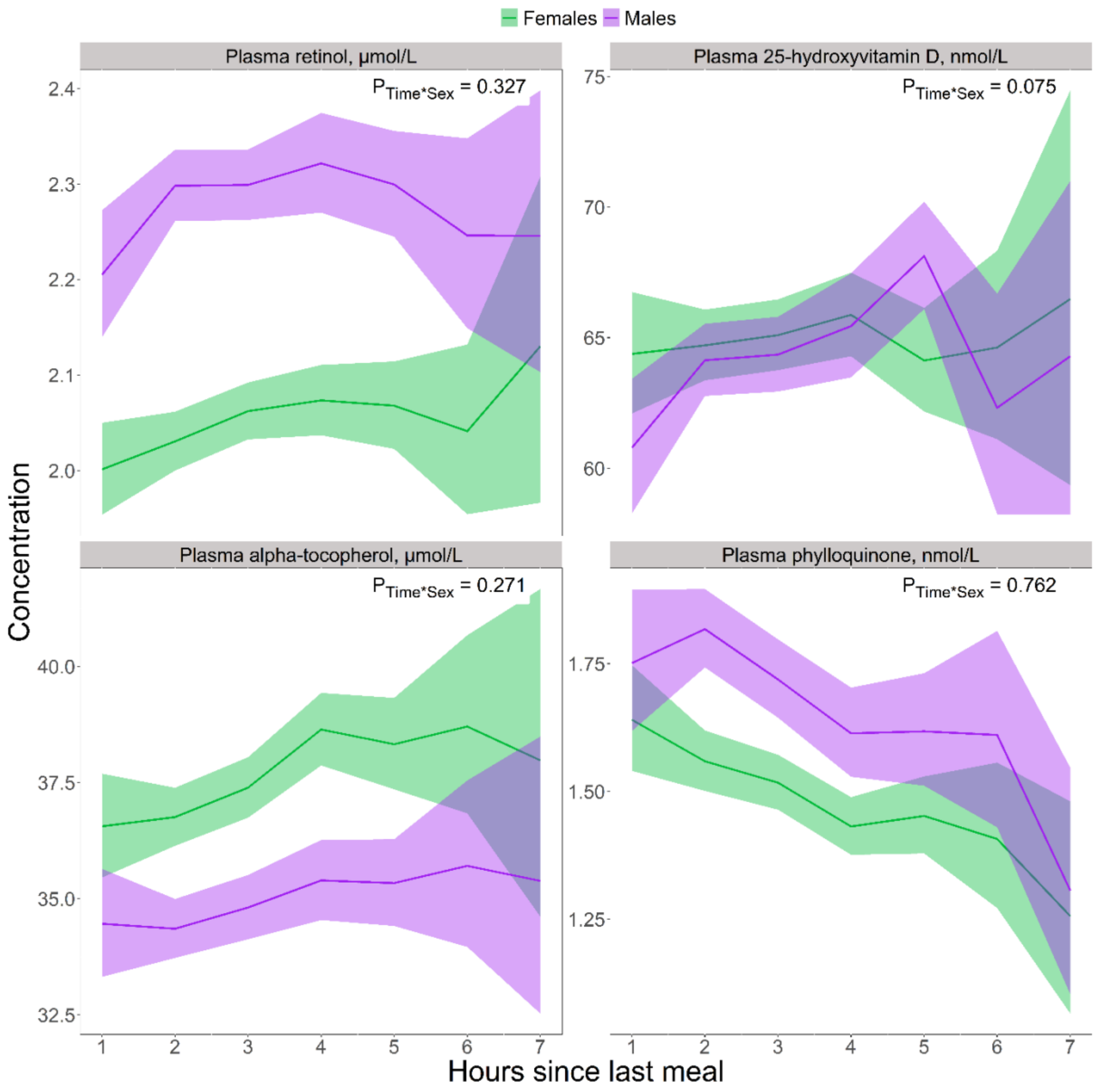

Supplementary Figure 10. The concentration of lipid-soluble vitamins as a function of time since the last meal in males (*n* = 2541) and females (*n* = 3293) in the Hordaland Health Study 1997-1999. The solid line represents the geometric mean, while the colored shaded area represent the 95% geometric confidence intervals. Note that the origin of the y-axis ≠ 0, and the y-axes are scales to be compatible with the metabolite concentration ranges. An overview of the number of observations at each timepoint is provided in Supplementary Table 4 and 5.

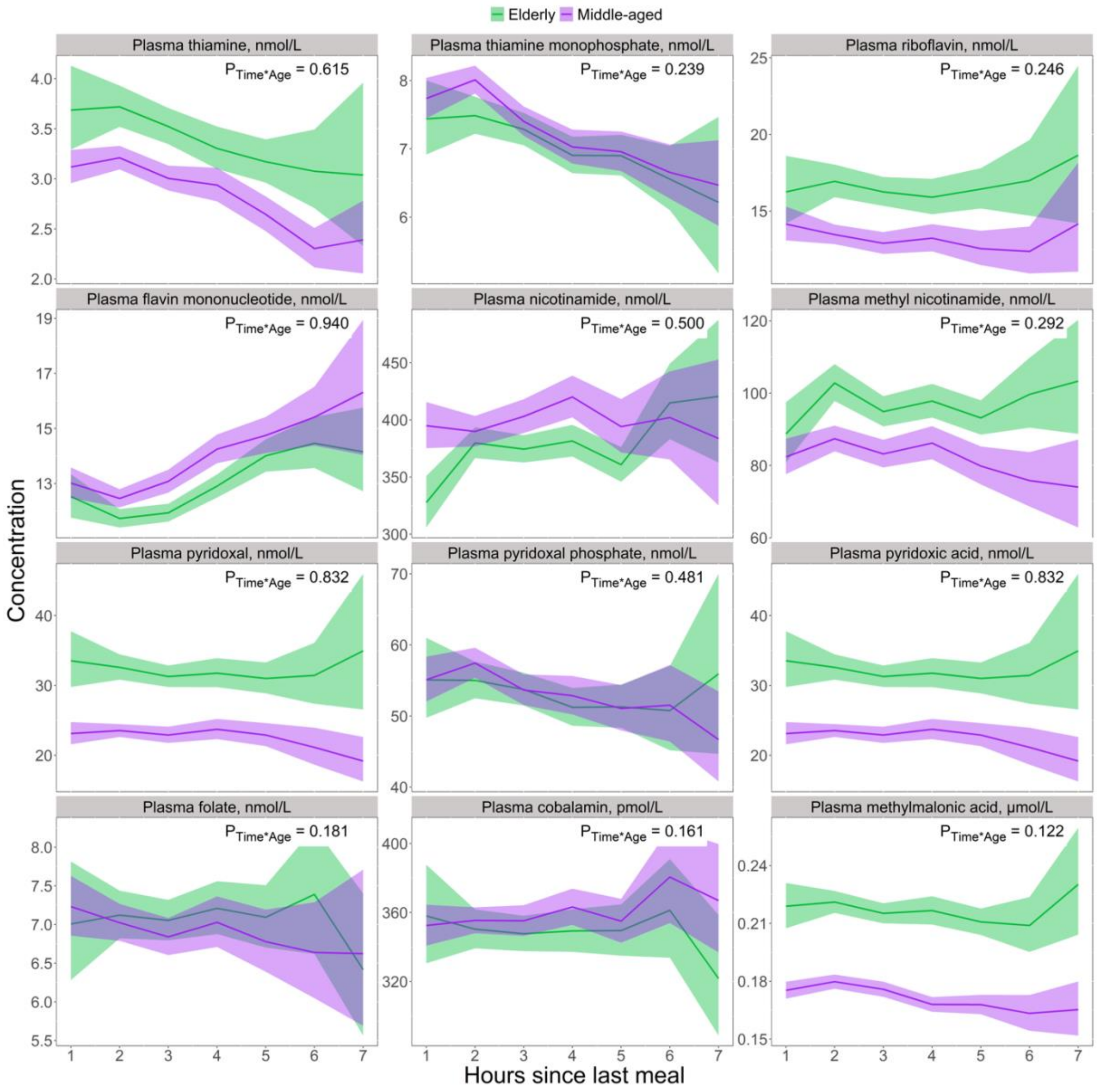

Supplementary Figure 11. The concentration of B-vitamin biomarkers as a function of time since the last meal in the middle-aged (*n* = 2960) and the elderly group (*n* = 2874) in the Hordaland Health Study 1997-1999. The solid line represents the geometric mean, while the colored shaded area represent the 95% geometric confidence intervals. Note that the origin of the y-axis ≠ 0, and the y-axes are scales to be compatible with the metabolite concentration ranges. An overview of the number of observations at each timepoint is provided in Supplementary Table 4 and 5.

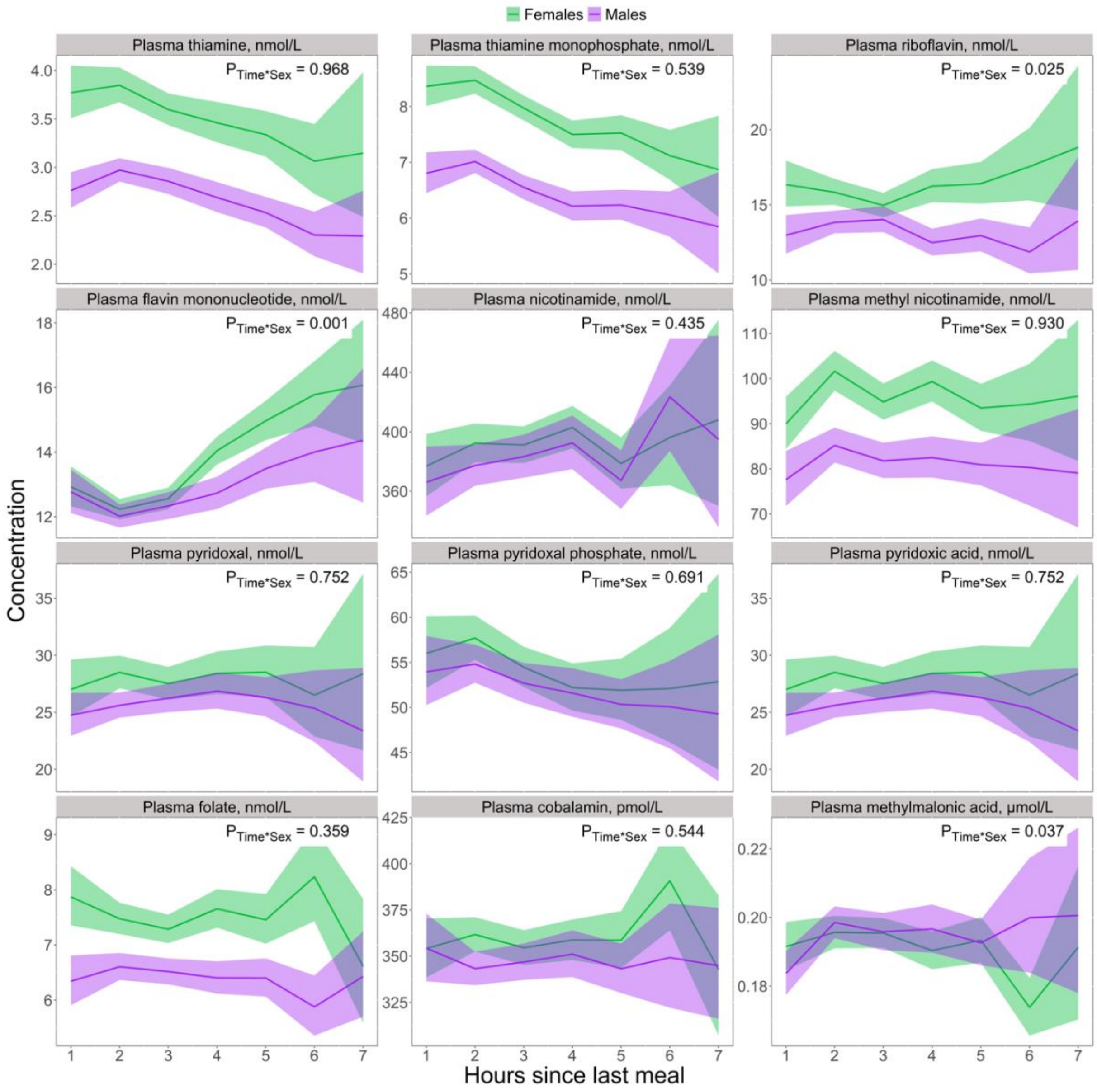

Supplementary Figure 12. The concentration of B-vitamin biomarkers as a function of time since the last meal in males (*n* = 2541) and females (*n* = 3293) in the Hordaland Health Study 1997-1999. The solid line represents the geometric mean, while the colored shaded area represent the 95% geometric confidence intervals. Note that the origin of the y-axis ≠ 0, and the y-axes are scales to be compatible with the metabolite concentration ranges. An overview of the number of observations at each timepoint is provided in Supplementary Table 4 and 5.
